# Investigating the mechanism of ShuFeng JieDu capsule for the treatment of novel Coronavirus pneumonia (COVID-19) based on network pharmacology

**DOI:** 10.7150/ijms.46378

**Published:** 2020-09-12

**Authors:** Xiao Chen, Yun-Hong Yin, Meng-Yu Zhang, Jian-Yu Liu, Rui Li, Yi-Qing Qu

**Affiliations:** 1 Department of Pulmonary and Critical Care Medicine, Qilu Hospital, Cheeloo College of Medicine, Shandong University, Jinan, China.; 2 Department of Pulmonary and Critical Care Medicine, Qilu Hospital, Shandong University, Jinan, China.; 3 Department of Respiratory Medicine, Tai'an City Central Hospital, Tai'an, China.

**Keywords:** ShuFeng JieDu capsule, Novel Coronavirus Pneumonia, network pharmacology, mechanism, pathway, candidate genes

## Abstract

ShuFeng JieDu capsule (SFJDC), a traditional Chinese medicine, has been recommended for the treatment of COVID-19 infections. However, the pharmacological mechanism of SFJDC still remains vague to date. The active ingredients and their target genes of SFJDC were collected from TCMSP. COVID-19 is a type of Novel Coronavirus Pneumonia (NCP). NCP-related target genes were collected from GeneCards database. The ingredients-targets network of SFJDC and PPI networks were constructed. The candidate genes were screened by Venn diagram package for enrichment analysis. The gene-pathway network was structured to obtain key target genes. In total, 124 active ingredients, 120 target genes of SFJDC and 251 NCP-related target genes were collected. The functional annotations cluster 1 of 23 candidate genes (CGs) were related to lung and Virus infection. RELA, MAPK1, MAPK14, CASP3, CASP8 and IL6 were the key target genes. The results suggested that SFJDC cloud be treated COVID-19 by multi-compounds and multi-pathways, and this study showed that the mechanism of traditional Chinese medicine (TCM) in the treatment of disease from the overall perspective.

## Introduction

Since December 2019, a novel coronavirus pneumonia (NCP) caused by new coronavirus (SARS-COV-2) has been prevalent in China and other countries, such as United States and Korea 1-3. WHO named this novel coronavirus pneumonia COVID-19 on February 11, 2020 4 and there was a total of 20 million reported cases of COVID-19 globally and 750,000 deaths as of August 10, 2020 5.

Its transmission route is mainly through respiratory droplets, but also through contact transmission, which has the characteristics of rapid spread, strong infectivity and general susceptibility of various groups of people. COVID-19 mild patients present with fever, fatigue, dry cough and other symptoms, whereas severe patients can appear with dyspnea, acute respiratory distress syndrome (ARDS) or septic shock and other symptoms. There is no special drug at present 6,7.

The treatment of COVID-19 mainly consisted of bed rest; intensive supportive treatment; oxygen therapy; antiviral therapy; antimicrobial therapy and Chinese medicine treatment. Critical cases need respiratory support (high flow nasal oxygen therapy, non-invasive ventilator or invasive mechanical ventilator); circulatory support for critically ill patients; plasma treatment from recovered patients and immunotherapy 8,9. Most of the infectious diseases caused by virus belong to the category of "plague" in ancient Chinese traditional medicine, which is caused by many evil spirits invading the body 10. The traditional medicine, including traditional Chinese medicine (TCM), has a good therapeutic effect on it 11,12. The Health and Health Commission of China and the State Administration of traditional Chinese Medicine in the "circular on the issuance of a new type of coronavirus infection pneumonia diagnosis and treatment program (version 5)" requested to strengthen the integration of Chinese and western medicine, and recommended a number of proprietary Chinese medicine in the process of diagnosis and treatment 13. On the basis of the national plan and in accordance with the principle of "three conditions and conditions", local prevention and control projects have also been successively issued according to local conditions 14. Recommended Chinese medicines include MaXing ShiGan Tang, QingFei PaiDu Tang, HuoXiang ZhengQi Capsules, JinHua QingGan Granules, LianHua QingWen Capsules or ShuFeng JieDu capsule 8. One clinical study showed that LianHua QingWen could improve the symptoms of COVID-19 patients and shorten the course of disease 15. A retrospective analysis study showed that the time of disappearance of clinical symptoms, recovery of body temperature, average length of stay in the integrated Chinese and western medicine treatment group (34) was significantly lower than that of the western medicine group (18) among the 52 COVID-19 patients 16.With QingFei PaiDu Tang combined with western medicine to treat the COVID-19 could significantly improve the patient's symptoms and achieved better results 17.

ShuFeng JieDu capsule (SFJDC) is a traditional Chinese medicine used to treat influenza in China 18. SFJDC is composed of Polygoni Cuspidati Rhizoma Et Radix (PCRR), Forsythiae Fructus (FF), Isatidis Radix (IR), Herba Patriniae (HP), Phragmitis Rhizoma (PR), Verbenae Herb (VH), licorice (I), Radix Bupleuri (RB) (**Table 1**). SFJDC has antiviral, anti-inflammatory, antipyretic and immune regulatory effects 19. SFJDC was commonly used for upper respiratory tract infection, pulmonary infection, AECOPD and other disease 20.This drug now is also recommended for the treatment of COVID-19 infections in the latest Diagnosis and Treatment of Pneumonia Caused by COVID-19 (version 5) 13,21. Currently, SFJDC is recommended in the Diagnosis and Treatment of Pneumonia Caused by COVID-19 in 5 provinces and cities 22.

Network pharmacology is a new discipline based on the theory of system biology, which analyzes the biological systems and selects specific signal nodes for multi-target drug molecular design. Network pharmacology emphasizes the multi-pathway regulation of signaling pathways and the regulation of multi-component, multi-target, multi-pathway, linking active components in traditional chinese medicine with target genes from molecular and biological aspects 23. Network pharmacology will help to understand the relationship among ingredients, genes and diseases and is suitable for the study of complex TCM or TCM compounds. The potential mechanism of preventing COVID-19 by HuoXiang ZhengQi oral solution was realized by network pharmacology and molecular docking 24. The research group Jing Zhao elucidated the mechanism of QingFei PaiDu Tang in the treatment of COVID-19 using network pharmacology 25. SFJDC could be efficacious for COVID-19, but active incredients, target genes and putative mechanism are not known.

**Figure 1:**
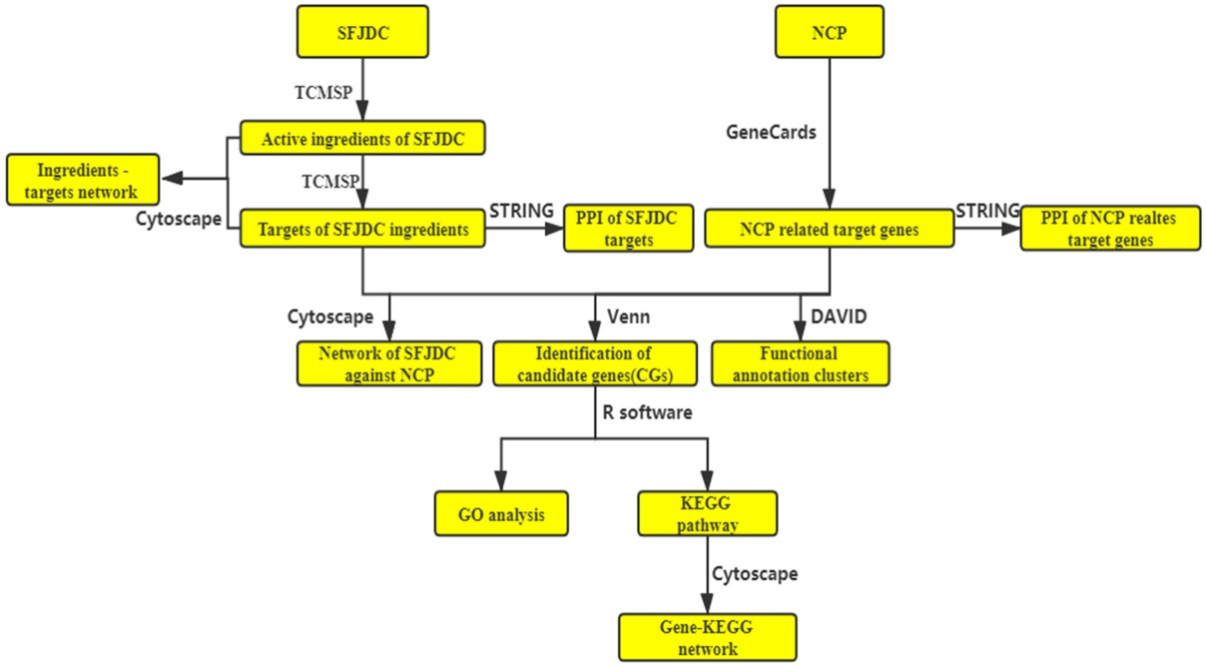
The flowchart of the whole manuscript base on network pharmacology.

In the present study, the network pharmacological was used to investigate the possible mechanism and target of SFJDC in the treatment of COVID-19. COVID-19 is a type of Novel Coronavirus Pneumonia (NCP). The active ingredients and their target genes of SFJDC were collected from TCMSP. NCP-related target genes were collected from GeneCards database. The putative mechanism of SFJDC against NCP were analyzed by GO and KEGG pathway. The flowchart of network pharmacology was shown in **Figure 1**. The study provided possible theoretical reference for SFJDC in the prevention and treatment of COVID-19.

## Materials and Methods

### Screening of active Ingredients in SFJDC

We identified the active ingredients of SFJDC from Traditional Chinese Medicine Systems Pharmacology Database and Analysis Platform (TCMSP http://tcmspw.com/tcmsp.php) 26. TCMSP is a unique herbal pharmacology platform that captures the relationship between drugs, target genes and diseases. The database includes the detection of natural compounds such as chemical, target and drug target networks. ADME is pharmacokinetics, which refers to the absorption, distribution, metabolism and excretion of exogenous chemicals by myosome. The four key parameters of ADME were blood-brain barrier (BBB), oral bioavailability (OB), Caco-2 permeability (Caco-2) and drug-likeness (DL) 27. In this study select candidate compounds which has OB≥30%, DL≥ 0.18, Caco-2≥-0.4, BBB≥-0.3.Then we sorted out each active ingredient for identification of targets.

### Identification of SFJDC putative target genes

This study used the TCMSP platform to obtain the putative target genes of active ingredients of SFJDC. The Uniprot (https://www.uniprot.org/) 28 database provides a comprehensive, high quality and freely available source of protein sequence and function information. The putative target information corresponding to the active ingredients were input into UniProt database to obtain the standard name of the action target genes.

### Screening of NCP related targets

COVID-19 is a type of Novel Coronavirus Pneumonia (NCP). So We collected NCP related targets from GeneCards (https://www.genecards.org/), which is a searchable, integrative database that provides comprehensive, user-friendly information on all annotated and predicted human genes 29. The key word “Novel Coronavirus Pneumonia” was used in the GeenCards database.

### PPI (Protein-Protein Interaction) network construction of SFJDC putative and NCP related target genes

The PPI network of SFJDC putative and NCP related targets would be obtained from STRING (https://string-db.org/ ver11.0, update Jan 2019) 30. Active interaction sources were set as follows: Textmining, Co-expression, Neighborhood, Experiments, Databases, Gene Fusion and Co-occurrence. The required minimum interaction score was set at 0.4 in PPI network of SFJDC related targets, PPI network of NCP was set at 0.9. The barplot were generated by the R software (https://www.r-project.org/ver 3.6.2) based on counts value.

### Construction of SFJDC ingredient-target network

Perl (https://www.perl.org/get.html) is a programming language suitable for writing simple scripts as well as complex applications. We used Strawberry Perl 5.30.1.1 to prepare the ingredient-target network. Cytoscape is a universal open source software for large-scale integrated development of molecular interaction networks working data. Then the ingredients-targets network of SFJDC was constructed using Cytoscape 3.7.2 software 31.

### PPI network construction of SFJDC against NCP

In order to reveal the mechanism of SFJDC against NCP, a PPI network was constructed by the BisoGenet client which is a Cytoscape plugin was used to visualize. In this plugin, Protein-protein interactions information is taken from the DIP, BIOGRID, HPRD, INTACT, MINT, BIND 32. CytoNCA is a Cytoscape plugin integrating calculation, evaluation and visualization analysis for multiple centrality measure measures including Betweenness Centrality (BC), Degree Centrality (DC), Colseness Centrality (CC), Local average connectivity-based method (LAC), Eigenvector Centrality (EC) and Network Centrality (NC) 33.

### Identification of candidate genes (CGs) and enrichment analysis of CGs

The CGs were filtered with R software using the Venn Diagram package (https://cran.r-project.org/web/packages/VennDiagram/index.html). The CGs would be used for Gene Ontology (GO) analysis (including biological processes (BP), molecular functions (MF), and cellular components (CC)) and Kyoto Encyclopedia of Genes and Genomes (KEGG) pathways. GO and KEGG pathway analyses results were processed by the “enrichplot” (http://www.bioconductor.org/packages/release/bioc/html/enrichplot.html) “clusterProfiler” (http://www.bioconductor.org/packages/release/bioc/html/clusterProfiler.html) and “ggplot2” packages by R software. A *P* value of less than 0.05 was used regarded as statistically significant. At the same time, we input CGs into DAVID (https://david.ncifcrf.gov/) for functional enrichment analysis to obtain disease clustering.

### Construction of gene-pathway network

KEGG pathways that had significant changes of *P<*0.05 were further analyzed. The genes that significantly regulated pathways for gene-pathway network construction. The key target genes of SFJDC against NCP were screened by gene-pathway network.

## Results

### The active ingredients of each herb contained in SFJDC

One hundred and thirty-seven active ingredients were screened out of TCMSP based on ADME, 4 in PCRR, 17 in FF, 25 in IR, 9 in HP, 7 in PR, 7 in VH, 1 in I, 67 in RB and 13 of which were repeated. Finally, 124 candidate active components of each herb contained in SFJDC were screened for further analysis after removing duplation (**Table 2**).

### Putative target genes of each herb in SFJDC and NCP related target genes

The 124 candidate active components were imported into TCMSP database and Uniport database to identify the Putative target genes of each herb in SFJDC. One hundred and ten components were finally selected after removing 14 ingredients which did not link to any target genes. The target genes of 110 compounds were collected. 1705 genes were identified, 103 in PR, 209 in IR, 65 in HP, 1052 in RB, 75 in PCRR, 173 in FF and 27 in I. There were 1585 genes of the eight herbs overlapped, which was suggestive of potential interaction between the compounds of SFJDCA in the course of treatment. A total of 120 genes were identified after removing duplation (**Table 3**). And 251 NCP related target genes were identified from Gene Cards database (**Table 4**).

**PPI network of SFJDC putative and NCP related target genes**

**Figure 2:**
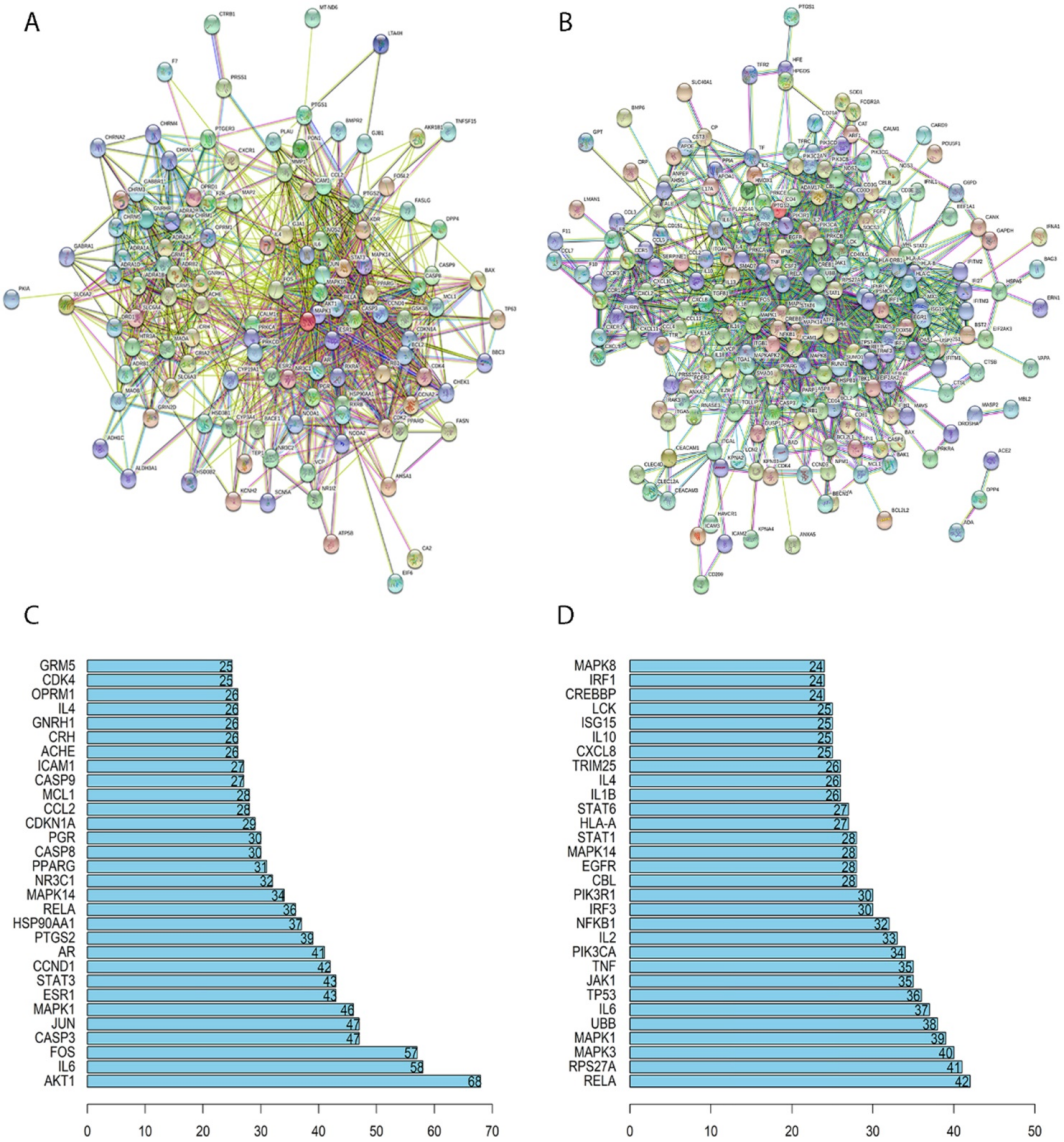
PPI network of SFJDC putative and NCP related target genes and the Barplot of PPI. (**A**) PPI network of SFJDC putative target genes. (**B**) PPI network of NCP related target genes. (**C**) Barplot showing the significant genes in PPI network of SFJDC. (**D**) Barplot showing the significant genes in PPI network of NCP. PPI, protein-protein interaction; SFJDC: ShuFeng JieDu capsule; NCP: Novel Coronavirus Pneumonia.

In this study, we constructed the PPI network of SFJDC putative and NCP related target genes separately. The network of SFJDC putative target genes which minimum interaction score was set at 0.4 contained 119 nodes and 1108 edges which indicated the target genes interactions after removing the discrete points (**Figure 2A**). According the PPI network, the top thirty genes were listed in **Figure 2B.** After hiding the discrete points, NCP-related target genes PPI network contained 248 nodes and 1235 edges (**Figure 2C**). Similarly, the first 30 related genes were shown in **Figure 2D.**

### SFJDC ingredient-target network analysis

**Figure 3:**
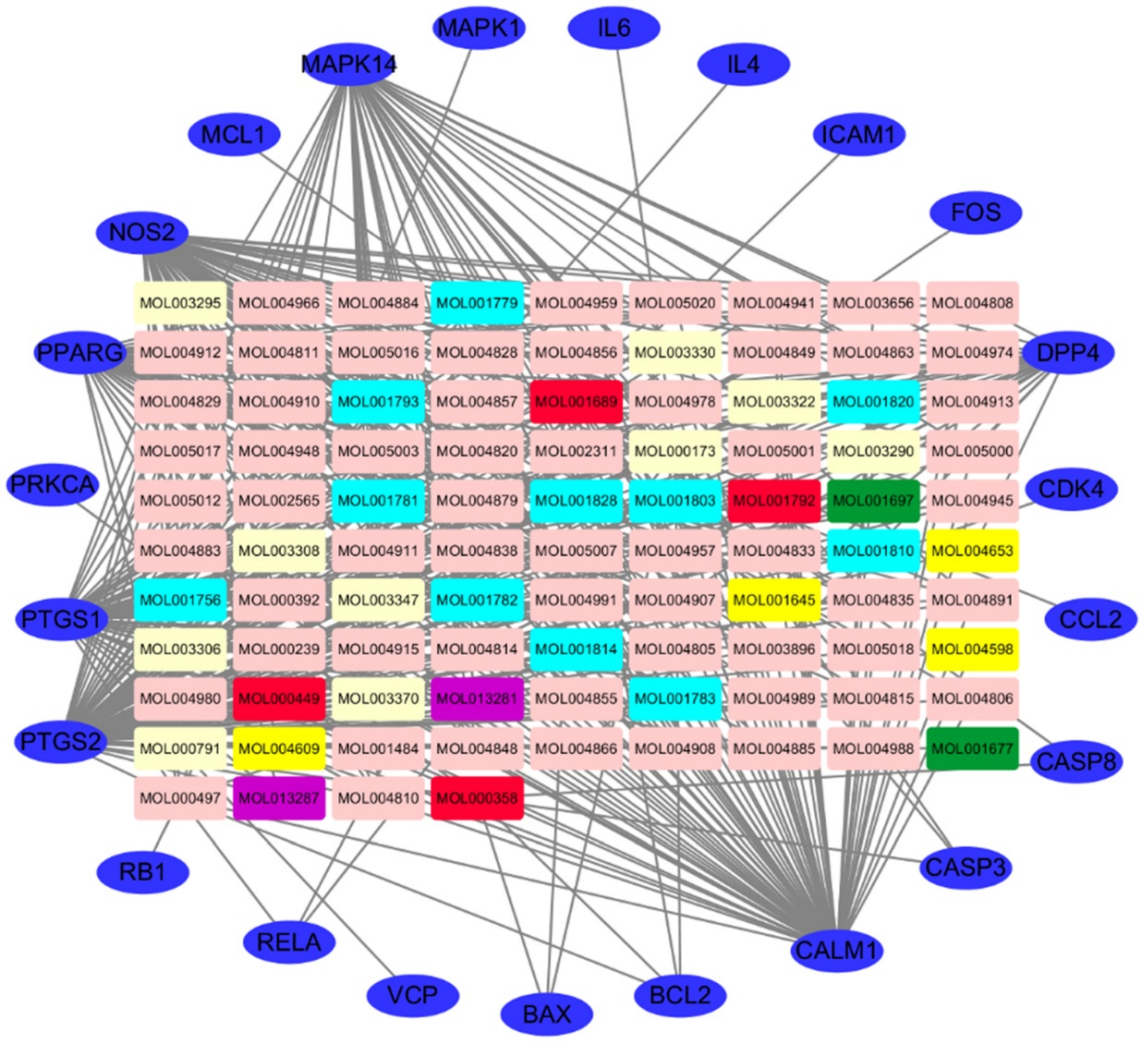
Ingredient-target network of SFJDC. The blue ovals represent target genes; the green, light blue, yellow, pink, purple and light yellow rectangulars represent the ingredients from PR, IR, HP, RB, PCRR, FF; the red rectangulars represent the ingredients from mlti-herb. PR: Phragmitis Rhizoma; IR: Isatidis Radix; HP: Herba Patriniae; RB:Radix Bupleuri; PCRR: Polygoni Cuspidati Rhizoma Et Radix; FF: Forsythiae Fructus.

The ingredient-target network of SFJDC was constructed using the screened ingredients and their targets as shown in **Figure 3.** The network contained 117 nodes and 419 edges which indicated the compound-target genes interaction. A median of 110 candidate compouds was 5 degrees which indicating that most compounds of SFJDC were affected by multiple target genes. The top three effective ingredient according were Wogonin, licochalcone a and acacetin. Wogonin, licochalcone a and acacetin have 42, 30 and 23 target genes, respectively. And the OB of Wogonin, licochalcone a and acacetin were 30.68, 40.79 and 34.97%, respectively. Hence, they might be the crucial effective compounds of SFJDC according the network.

### PPI network analysis of SFJDC against NCP

**Figure 4:**
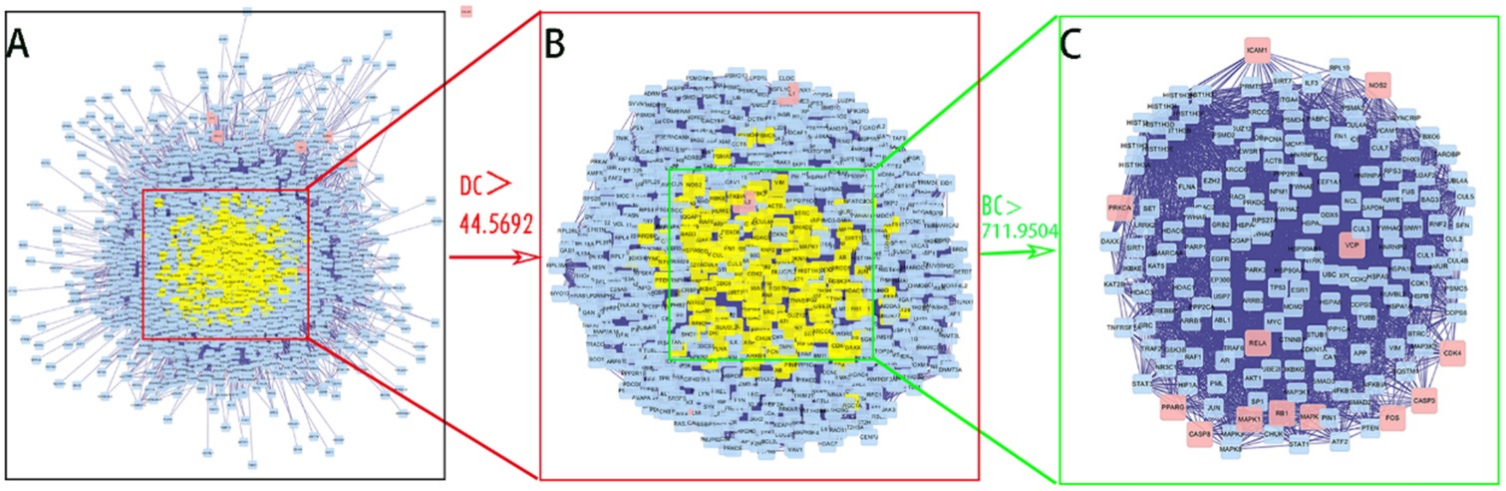
PPI network of SFJDC against NCP. (**A**)The whole network of SFJDC against NCP contained 2,407 nodes and 53,639 edges. (**B**) A subnetwork of significant genes from A consisted of 766 nodes and 28872 edges. (**C**) PPI network of more significant genes from B with 169 nodes and 4238 edges. BC: Betweenness Centrality; DC: Degree Centrality.

PPI network of SFJDC against NCP were visualized using Cytoscape software. The network contained 2407 nodes and 53639 edges was shown in **Figure 4A**. The average degree of all nodes was 44.5692 and we selected the nodes with more than 44.5692 degrees as significant genes. A subnetwork of significant genes for SFJDC against NCP was constructed which consisted of 766 nodes and 28872 edges (**Figure 4B**). The average value of BC was 711.9504. The significant genes were further screened and a new network was constructed with 169 nodes and 4238 edges (**Figure 4C**). 169 genes were eventually identified for SFJDC against NCP including 156 other human genes and 13 target genes.

### Identification of candidate genes (CGs) and Enrichment analysis of CGs

**Figure 5:**
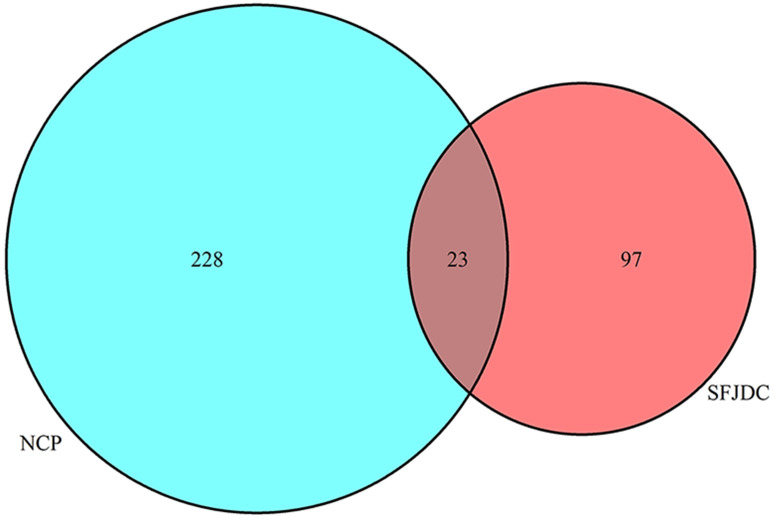
Twenty-three overlapping genes between SFJDC and NCP.

**Figure 6:**
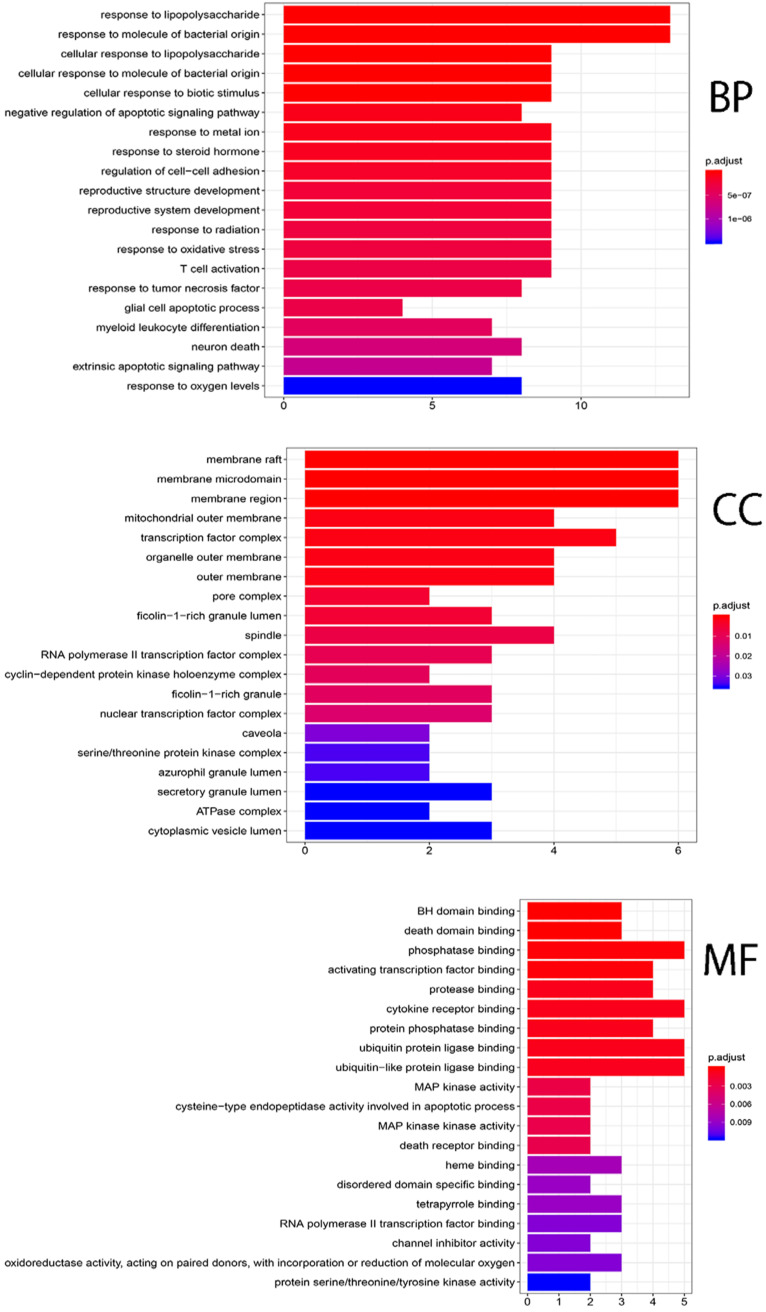
Gene ontology terms of CGs. The top 20 GO functional terms were selected (*P*<0.05). BP: biological processes; CC: cellular components; MF: molecular functions.

Twenty-three candidate genes (CGs) were identified by using the VennDiagram package (**Figure 5**). Then R software was used to perform GO and KEGG pathway analysis of the CGs. GO of CGs was analyzed based on BP, CC, MF. 1215 GO terms were significantly enriched (*P<*0.05), 1148 in BP, 28 in CC, 39 in MF. Top 20 terms were shown in **Figure 6.** The data of top 20 GO analysis were listed in **Table 5.** Based on these GO terms data, we found that most significantly terms were response to lipopolysaccharide, response to molecule of bacterial origin, membrane raft, membrane microdomain, BH domain binding and death domain binding, suggested that SFJDC could treat NCP with multiple synergies.

**Figure 7:**
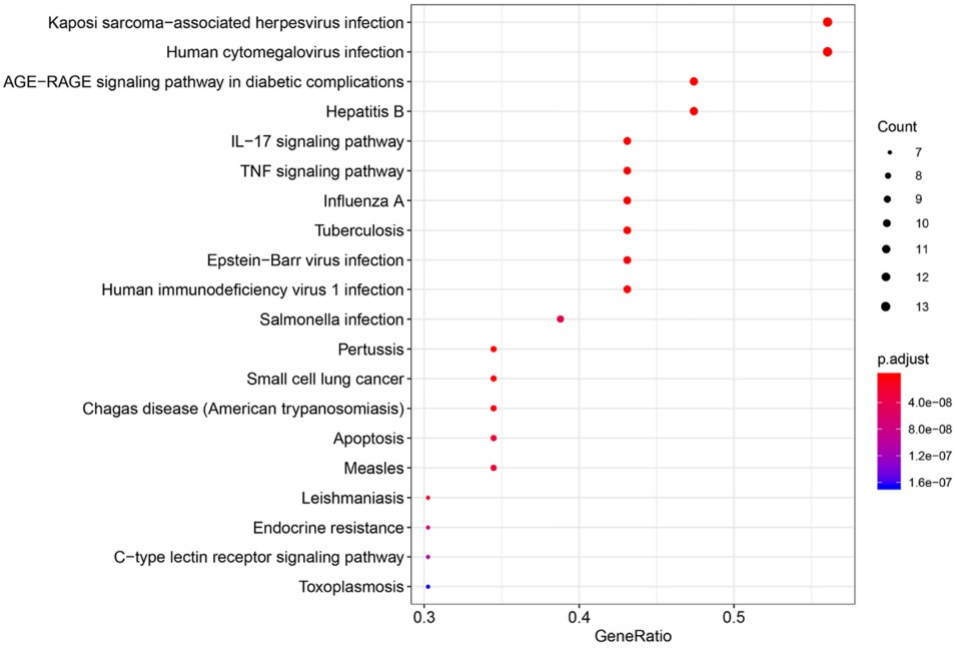
KEGG pathway enrichment of CGs. The top 20 pathways were identified. Color represented *P* value and size of the spot represented count of genes.

The pathways that were significantly affected by SFJDC in the process of treating NCP were identified by KEGG pathway. 110 KEGG pathways were significantly enriched (*P<*0.05). Top twenty pathways were shown in** Figure 7**, color represented *P* value and size of the spot represented count of genes. Based on the analysis of KEGG pathway data (**Table 6**), the top five pathways such as Kaposi sarcoma-associated herpesvirus infection, AGE-RAGE signaling pathway in diabetic complications, Human cytomegalovirus infection, IL-17 signaling pathway and Hepatitis B, might be the core pharmacological mechanism of SFJDC for NCP.

In this study, we chose the functional annotation clustering and set the classification stringency as high in DAVID. A total of 20 functional annotation clusters were obtained (**Table 7**). Annotation Cluster 1 (enrichment score 6.04) contains three categories: Asthma, Bronchiolitis Viral, Respiratory Syncytial Virus Infections, respiratory syncytial virus bronchiolitis, and all of them were lung related diseases and Virus infection disease.

### Gene-pathway network analysis

**Figure 8:**
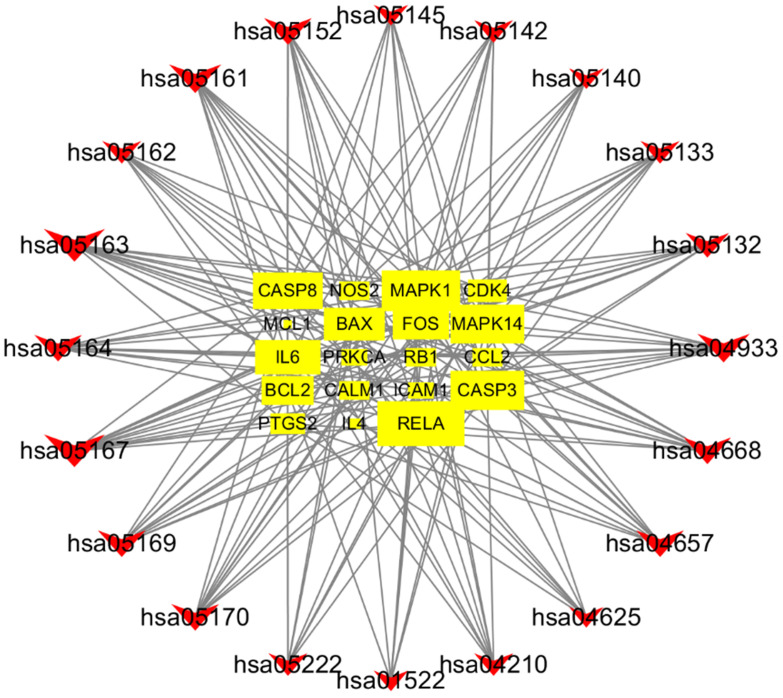
Gene-pathway network of SFJDC against NCP. The V shapes represented pathway and the squares represent target genes in the network.

The construction of gene-pathway network is based on significant enrichment pathway and regulated these ways, which was shown in **Figure 8.** The V shapes represented pathway and the squares represent target genes in the network. The network showed that RELA was the core target gene which had largest degree. Other five genes also had larger degree such as MAPK1, MAPK14, CASP3, CASP8 and IL6. They might be the key target genes using SFJDC in the process of treating NCP. All of the above analysis could reveal a new strategy for drug development on NCP.

## Discussion

The theory of TCM has been formed and developed for thousands of years in China. In China, TCM has a good therapeutic effect on COVID-19, which has been written into the diagnosis and treatment guidelines. The guideline points out that the combination of traditional Chinese and western medicine should be strengthened in the treatment process 34. SFJDC is a traditional Chinese medicine, mainly used to treat upper respiratory tract infections, such as influenza, sore throat, mumps, streptococcus, etc. 21. Now, SFJDC has become an effective drug for the treatment of COVID-19 35. In recent years, the research on Chinese medicine prescriptions has developed to the level of effective parts, components, components. Network pharmacology can better understand and demonstrate the interaction between multi-component multi-target and disease 36. This study aims to analyze the active components and potential mechanism of SFJDC in the treatment of COVID-19 through network pharmacology.

In the present study, the ingredients-targets network of SFJDC was constructed using 110 ingredients and 120 targets. The network contained 117 nodes and 419 edges which indicated the compound-target genes interaction. The results showed that most compounds of SFJDC were affected by multiple target genes, such as Wogonin, licochalcone a and acacetin acted on 42, 30 and 23 target genes, respectively. Various compounds of SFJDC may have the same targets to achieve synergy. Wogonin, a naturally occurring flavonoid, has been shown to multi-activity, such as anti-inflammatory, anti-fibrosis, anti-cancer and chondroprotective properties 37. Study showed that wogonin had an anti-infulenza activity by modulation of AMPK pathway 38. Licochalcone a, a flavonoid extracted from licorice toot, was known for its anti-inflammatory, anti-cancer, anti-oxidative and anti-bacterial bioactivity 39. Acacetin, a flavone compound, played an important role in anti-inflammatory and anti-peroxidative 40.

In addition, they have high OB and acacetin from 2 herbs (PR, IR) of SFJDC. The three main ingredients were anti-inflammatory and COVID-19 caused by a series of inflammatory storms. Hence, they might be the crucial effective compounds of SFJDC according the network.

PPI network of SFJDC against NCP were visualized using Cytoscape software to obtain the candidate target genes. In order to obtain the more accurate genes, two parameters including DC and BC were used to screen nodes and structure a new network. 169 genes were eventually identified for SFJDC against NCP including 156 other human genes and 13 target genes.

Twenty-three candidate genes (CGs) were identified by using the VennDiagram package. CGs were enriched in BP, CC, MF by GO enrichment analysis. Based on GO terms data, we found that some terms were response to lipopolysaccharide or bacterial origin, membrane raft, membrane microdomain, BH domain binding and cytokine receptor binding. COVID-19 infections leaded to a strong immune response and inflammatory storm in which a large number of cytokines were activated, so SFJDC might regulate COVID-19 through the above biological processes.

SFJDC, as a TCM formula, has multi-component, multi-target-gene, multi-pathway. In the present study, 110 KEGG pathways were significantly enriched. Seven of the top 20 pathways were associated with viral infection including Kaposi sarcoma-associated herpesvirus infection, Human cytomegalovirus infection, Hepatitis B, Influenza A, Epstein-Barr virus infection, Human immunodeficiency virus 1 infection and Measles, and three were associated with lung disease contained tuberculosis, pertussis and small cell lung cancer. Multiple targets of SFJDC may also inhibit the activation of cytokines and reduce inflammation by regulating cytokine pathways, such as IL-17 signaling pathway and TNF signaling pathway. In this study, we obtained 20 functional annotation clusters through DAVID. Annotation Cluster1 including Asthma, Bronchiolitis Viral, Respiratory Syncytial Virus Infections, respiratory syncytial virus bronchiolitis were lung related diseases and Virus infection disease.

Gene-pathway network was constructed to the core and key target genes. The network showed that RELA had largest degree, was the core target gene. Other top five genes such as MAPK1, MAPK14, CASP3, CASP8 and IL6 might be the key target genes. The pathophysiological process of Severe Acute Respiratory Syndrome-Coronavirus-2 (SARS-COV-2) infection is similar to that of SARS-CoV infection, with a strong inflammatory response. The SARS-COV-2 virus mainly targets respiratory epithelial cells, alveolar epithelial cells, vascular endothelial cells and pulmonary macrophages, all of which express Angiotensin converting enzyme 2 (ACE2) receptor, triggering the generation of pro- inflammatory cytokines and chemokines (including IL-6, TNF, IL-10 and MCP1) 41. The NF-kB family member RELA is a widely expressed and effective transcriptional activator that activates the expression of many inflammatory through exposure to pathogens and inflammatory cytokines 42. RELA may play an important role in the infection of COVID-19. MAPK1 and MAPK14 are members of the MAPK family, which can regulate multiple cellular processes, such as response to oxidative stress, anti-inflammatory, immune response, apoptosis and cell proliferation 43. Joseph et al showed SASR-CoV-2 could induce severe inflammation by directly activating p38 MAPK pathway and many p38 MAPK inhibitors are in the clinical stage and should be considered for clinical trial for severe COVID-19 infection 44. CASP3 and CASP8, a family of cysteine-dependent proteases, play an important role in these events through activation of other apoptotic proteins mediated by proteolysis and cleavage of nuclear proteins 45. In Krahling's study, infection of 293/ACE2 cells with SARS-CoV activated apoptosis-associated events, such as caspase3, caspase 8^46^. Therefore, we conclude that CASP3 and CASP8 may be activated and play an important role in the pathophysiological process of COVID-19. Higher plasma level of IL-6 was found in ICU patients with COVID-19^47^. Tocilizumab, a recombinant humanized anti-human IL-6 receptor monoclonal antibody, improved the clinical outcome in 20 severe and critical COVID-19 patients and is an effective treatment to reduce mortality 48.

It has been clinically confirmed that SFJDC is effective in the treatment of COVID-19. Wang et al shown that conventional treatment combined with SFJDC treatment for 4 cases of COVID-19 patients could significantly improve symptoms and promote viral negative conversion 49. Another study including 70 COVID-19 patients found that SFJDC combined with Arbidol for COVID-19 compared with single using Arbidol could significantly shorten the time of clinical symptoms improvement and COVID-19 negative conversion 50.

To summarise, the compound and targets of SFJDC were systematically studied by applying network pharmacology. Wogonin, licochalcone a and acacetin regulated the most targets associated with NCP. RELA, MAPK1, MAPK14, CASP3, CASP8 and IL6 were the core and key genes in the gene-network of SFJDC for the treatment of NCP. SFJDC regulated novel coronavirus pneumonia by multi-compound and multi-target, which provided theoretical support for SFJDC against COVID-19. More mechanism and roles require further clinical validation.

## Figures and Tables

**Table 1:** Herb composition of Shu Feng Jie Du Capsule (SFJDC)

English translation	Latin name	Chinese name
Hu-Zhang	Polygoni Cuspidati Rhizoma Et Radix	虎杖
Lian-Qiao	Forsythiae Fructus	连翘
Ban-Lan-Gen	Isatidis Radix	板蓝根
Chai-Hu	Herba Patriniae	柴胡
Bai-Jiang-Cao	Phragmitis Rhizoma	败酱草
Ma-Bian-Cao	Verbenae Herb	马鞭草
Lu-Gen	licorice	芦根
Gan-Cao	Radix Bupleuri	甘草

**Table 2:** The active ingredients of each herb contained in SFJDC

Mol ID	Molecule Name	OB (%)	Caco-2	BBB	DL	Source
MOL000173	wogonin	30.68	0.79	0.04	0.23	FF
MOL000211	Mairin	55.38	0.73	0.22	0.78	FF; RB
MOL000239	Jaranol	50.83	0.61	-0.22	0.29	RB
MOL000358	beta-sitosterol	36.91	1.32	0.99	0.75	PR; PCRR; IR; FF; VH
MOL000359	sitosterol	36.91	1.32	0.87	0.75	PR; IR; RB
MOL000392	formononetin	69.67	0.78	0.02	0.21	RB
MOL000449	Stigmasterol	43.83	1.44	1	0.76	PR; IR; HP; I; VH
MOL000497	licochalcone a	40.79	0.82	-0.21	0.29	RB
MOL000791	bicuculline	69.67	0.72	0.02	0.88	FF
MOL000953	CLR	37.87	1.43	1.13	0.68	IR
MOL001484	Inermine	75.18	0.89	0.4	0.54	RB
MOL001645	Linoleyl acetate	42.1	1.36	1.08	0.2	HP
MOL001663	(4aS,6aR,6aS,6bR,8aR,10R,12aR,14bS)-10-hydroxy-2,2,6a,6b,9,9,12a-heptamethyl-1,3,4,5,6,6a,7,8,8a,10,11,12,13,14b-tetradecahydropicene-4a-carboxylic acid	32.03	0.61	0.39	0.76	VH
MOL001676	Vilmorrianine C	33.96	0.59	0.14	0.22	PR
MOL001677	asperglaucide	58.02	0.28	-0.22	0.52	PR
MOL001689	acacetin	34.97	0.67	-0.05	0.24	PR; IR
MOL001697	Sinoacutine	63.39	0.72	0.36	0.53	PR
MOL001749	ZINC03860434	43.59	1.04	0.6	0.35	IR
MOL001755	24-Ethylcholest-4-en-3-one	36.08	1.46	1.22	0.76	IR
MOL001756	quindoline	33.17	1.5	0.99	0.22	IR
MOL001769	beta-sitosterol dodecantate	34.57	1.28	0.57	0.57	IR
MOL001771	poriferast-5-en-3beta-ol	36.91	1.45	1.14	0.75	IR
MOL001774	Ineketone	37.14	0.39	0.1	0.3	IR
MOL001779	Sinoacutine	49.11	0.7	0.39	0.46	IR
MOL001781	Indigo	38.2	0.83	0.02	0.26	IR
MOL001782	(2Z)-2-(2-oxoindolin-3-ylidene)indolin-3-one	48.4	0.85	-0.06	0.26	IR
MOL001783	2-(9-((3-methyl-2-oxopent-3-en-1-yl)oxy)-2-oxo-1,2,8,9-tetrahydrofuro[2,3-h]quinolin-8-yl)propan-2-yl acetate	64	0.39	-0.09	0.57	IR
MOL001792	DFV	32.76	0.51	-0.29	0.18	IR; RB
MOL001793	(E)-2-[(3-indole)cyanomethylene-]-3-indolinone	54.59	1.06	0.22	0.32	IR
MOL001800	rosasterol	35.87	1.28	0.89	0.75	IR
MOL001803	Sinensetin	50.56	1.12	0.04	0.45	IR
MOL001804	Stigmasta-5,22-diene-3beta,7alpha-diol	43.04	1.35	0.84	0.82	IR
MOL001806	Stigmasta-5,22-diene-3beta,7beta-diol	42.56	1.37	0.81	0.83	IR
MOL001810	6-(3-oxoindolin-2-ylidene)indolo[2,1-b]quinazolin-12-one	45.28	1.19	0.48	0.89	IR
MOL001814	(E)-3-(3,5-dimethoxy-4-hydroxy-benzylidene)-2-indolinone	57.18	0.69	0.16	0.25	IR
MOL001820	(E)-3-(3,5-dimethoxy-4-hydroxyb-enzylidene)-2-indolinone	65.17	0.28	-0.17	0.25	IR
MOL001828	3-[(3,5-dimethoxy-4-oxo-1-cyclohexa-2,5-dienylidene)methyl]-2,4-dihydro-1H-pyrrolo[2,1-b]quinazolin-9-one	51.84	0.81	0.03	0.56	IR
MOL002311	Glycyrol	90.78	0.71	-0.2	0.67	RB
MOL002565	Medicarpin	49.22	1	0.53	0.34	RB
MOL002773	beta-carotene	37.18	2.25	1.52	0.58	VH
MOL003281	20(S)-dammar-24-ene-3β,20-diol-3-acetate	40.23	0.93	0.28	0.82	FF
MOL003290	(3R,4R)-3,4-bis[(3,4-dimethoxyphenyl)methyl]oxolan-2-one	52.3	0.78	0.17	0.48	FF
MOL003295	(+)-pinoresinol monomethyl ether	53.08	0.69	0	0.57	FF
MOL003306	ACon1_001697	85.12	0.76	0	0.57	FF
MOL003308	(+)-pinoresinol monomethyl ether-4-D-beta-glucoside_qt	61.2	0.7	0.12	0.57	FF
MOL003315	3beta-Acetyl-20,25-epoxydammarane-24alpha-ol	33.07	0.75	0.24	0.79	FF
MOL003322	FORSYTHINOL	81.25	0.59	-0.08	0.57	FF
MOL003330	(-)-Phillygenin	95.04	0.75	0.07	0.57	FF
MOL003344	β-amyrin acetate	42.06	1.36	1.1	0.74	FF
MOL003347	hyperforin	44.03	0.87	0.4	0.6	FF
MOL003348	adhyperforin	44.03	0.93	0.58	0.61	FF
MOL003365	Lactucasterol	40.99	0.88	0.5	0.85	FF
MOL003370	Onjixanthone I	79.16	0.84	0.04	0.3	FF
MOL003656	Lupiwighteone	51.64	0.68	-0.23	0.37	RB
MOL003896	7-Methoxy-2-methyl isoflavone	42.56	1.16	0.56	0.2	RB
MOL004598	3,5,6,7-tetramethoxy-2-(3,4,5-trimethoxyphenyl)chromone	31.97	0.75	0.08	0.59	HP
MOL004609	Areapillin	48.96	0.6	-0.29	0.41	HP
MOL004624	Longikaurin A	47.72	0.08	0.09	0.53	HP
MOL004628	Octalupine	47.82	0.48	0.3	0.28	HP
MOL004644	Sainfuran	79.91	0.9	0.23	0.23	HP
MOL004653	(+)-Anomalin	46.06	0.46	0	0.66	HP
MOL004718	α-spinasterol	42.98	1.28	0.79	0.76	HP
MOL004805	(2S)-2-[4-hydroxy-3-(3-methylbut-2-enyl)phenyl]-8,8-dimethyl-2,3-dihydropyrano[2,3-f]chromen-4-one	31.79	1	0.25	0.72	RB
MOL004806	euchrenone	30.29	1.09	0.39	0.57	RB
MOL004808	glyasperin B	65.22	0.47	-0.09	0.44	RB
MOL004810	glyasperin F	75.84	0.43	-0.15	0.54	RB
MOL004811	Glyasperin C	45.56	0.71	0.07	0.4	RB
MOL004814	Isotrifoliol	31.94	0.53	-0.25	0.42	RB
MOL004815	(E)-1-(2,4-dihydroxyphenyl)-3-(2,2-dimethylchromen-6-yl)prop-2-en-1-one	39.62	0.66	-0.12	0.35	RB
MOL004820	kanzonols W	50.48	0.63	0.04	0.52	RB
MOL004828	Glepidotin A	44.72	0.79	0.06	0.35	RB
MOL004829	Glepidotin B	64.46	0.46	-0.09	0.34	RB
MOL004833	Phaseolinisoflavan	32.01	1.01	0.46	0.45	RB
MOL004835	Glypallichalcone	61.6	0.76	0.23	0.19	RB
MOL004838	8-(6-hydroxy-2-benzofuranyl)-2,2-dimethyl-5-chromenol	58.44	1	0.34	0.38	RB
MOL004848	licochalcone G	49.25	0.64	-0.04	0.32	RB
MOL004849	3-(2,4-dihydroxyphenyl)-8-(1,1-dimethylprop-2-enyl)-7-hydroxy-5-methoxy-coumarin	59.62	0.4	-0.23	0.43	RB
MOL004855	Licoricone	63.58	0.53	-0.14	0.47	RB
MOL004856	RBnin A	51.08	0.8	0.13	0.4	RB
MOL004857	RBnin B	48.79	0.58	-0.1	0.45	RB
MOL004863	3-(3,4-dihydroxyphenyl)-5,7-dihydroxy-8-(3-methylbut-2-enyl)chromone	66.37	0.52	-0.13	0.41	RB
MOL004866	2-(3,4-dihydroxyphenyl)-5,7-dihydroxy-6-(3-methylbut-2-enyl)chromone	44.15	0.48	-0.28	0.41	RB
MOL004879	Glycyrin	52.61	0.59	-0.13	0.47	RB
MOL004882	Licocoumarone	33.21	0.84	0.06	0.36	RB
MOL004883	Licoisoflavone	41.61	0.37	-0.27	0.42	RB
MOL004884	Licoisoflavone B	38.93	0.46	-0.18	0.55	RB
MOL004885	licoisoflavanone	52.47	0.39	-0.22	0.54	RB
MOL004891	shinpterocarpin	80.3	1.1	0.68	0.73	RB
MOL004907	Glyzaglabrin	61.07	0.34	-0.2	0.35	RB
MOL004908	Glabridin	53.25	0.97	0.36	0.47	RB
MOL004910	Glabranin	52.9	0.97	0.31	0.31	RB
MOL004911	Glabrene	46.27	0.99	0.04	0.44	RB
MOL004912	Glabrone	52.51	0.59	-0.11	0.5	RB
MOL004913	1,3-dihydroxy-9-methoxy-6-benzofurano[3,2-c]chromenone	48.14	0.48	-0.19	0.43	RB
MOL004915	Eurycarpin A	43.28	0.43	-0.06	0.37	RB
MOL004941	(2R)-7-hydroxy-2-(4-hydroxyphenyl)chroman-4-one	71.12	0.41	-0.25	0.18	RB
MOL004945	(2S)-7-hydroxy-2-(4-hydroxyphenyl)-8-(3-methylbut-2-enyl)chroman-4-one	36.57	0.72	-0.04	0.32	RB
MOL004948	Isoglycyrol	44.7	0.91	0.05	0.84	RB
MOL004957	HMO	38.37	0.79	0.25	0.21	RB
MOL004959	1-Methoxyphaseollidin	69.98	1.01	0.48	0.64	RB
MOL004966	3'-Hydroxy-4'-O-Methylglabridin	43.71	1	0.73	0.57	RB
MOL004974	3'-Methoxyglabridin	46.16	0.94	0.47	0.57	RB
MOL004978	2-[(3R)-8,8-dimethyl-3,4-dihydro-2H-pyrano[6,5-f]chromen-3-yl]-5-methoxyphenol	36.21	1.12	0.61	0.52	RB
MOL004980	Inflacoumarin A	39.71	0.73	-0.24	0.33	RB
MOL004985	icos-5-enoic acid	30.7	1.22	1.09	0.2	RB
MOL004988	Kanzonol F	32.47	1.18	0.56	0.89	RB
MOL004989	6-prenylated eriodictyol	39.22	0.4	-0.29	0.41	RB
MOL004991	7-Acetoxy-2-methylisoflavone	38.92	0.74	0.16	0.26	RB
MOL004996	gadelaidic acid	30.7	1.2	0.94	0.2	RB
MOL005000	RBnin G	60.44	0.78	0.23	0.39	RB
MOL005001	RBnin H	50.1	0.6	-0.14	0.78	RB
MOL005003	Licoagrocarpin	58.81	1.23	0.61	0.58	RB
MOL005007	Glyasperins M	72.67	0.49	-0.04	0.59	RB
MOL005012	Licoagroisoflavone	57.28	0.71	0.09	0.49	RB
MOL005016	Odoratin	49.95	0.42	-0.24	0.3	RB
MOL005017	Phaseol	78.77	0.76	-0.06	0.58	RB
MOL005018	Xambioona	54.85	1.09	0.52	0.87	RB
MOL005020	dehydroglyasperins C	53.82	0.68	-0.12	0.37	RB
MOL005229	Artemetin	49.55	0.81	-0.09	0.48	VH
MOL005503	Cornudentanone	39.66	0.47	0.09	0.33	VH
MOL008752	Dihydroverticillatine	42.69	0.56	0.11	0.84	VH
MOL013281	6,8-Dihydroxy-7-methoxyxanthone	35.83	0.68	0.1	0.21	PCRR
MOL013287	Physovenine	106.21	0.51	0.2	0.19	PCRR
MOL013288	Picralinal	58.01	0.23	-0.21	0.75	PCRR

**Table 3:** Putative target genes of each herb in SFJDC

Herb	Mol ID	Molname	Target genes
FF	MOL000173	wogonin	ADRB2 AHSA1 AKT1 AR BAX BBC3 BCL2 CALM1 CASP3 CASP9 CCL2 CCND1 CDK2 CDKN1A CHEK1 DPP4 EIF6 ESR1 FSD1 GABRA1 GSK3B HSP90AA1 IL6 IL8RA JUN KDR MAPK14 MCL1 MMP1 NOS2 PPARG PRKCD PRSS1 PTGER3 PTGS1 PTGS2 RELA RXRA SCN5A TEP1 TNFSF15 TP63
RB FF	MOL000211	Mairin	PGR
RB	MOL000239	Jaranol	AR CALM1 CDK2 CHEK1 DPP4 ESR2 HSP90AA1 NCOA2 NOS2 PRSS1 PTGS1 PTGS2 SCN5A
PR IR; PCRR; FF	MOL000358	beta-sitosterol	ADRA1A ADRA1B ADRB2 BAX BCL2 CASP3 CASP8 CASP9 CHRM1 CHRM2 CHRM3 CHRM4 CHRNA2 DRD1 GABRA1 HSP90AA1 JUN KCNH2 MAP2 NCOA2 OPRM1 PGR PON1 PRKCA PTGS1 PTGS2 SCN5A SLC6A4
PR IR RB	MOL000359	sitosterol	NCOA2 NR3C2 PGR
RB	MOL000392	formononetin	ACHE ADRA1A ADRB2 AR ATP5F1B CALM1 CCNA2 CDK2 CHEK1 CHRM1 DPP4 ESR1 ESR2 GSK3B HSD3B1 HSD3B2 HSP90AA1 HTR IL4 JUN MAOB MAPK14 ND6 NOS2 PKIA PPARG PPARG PRSS1 PTGS1 PTGS2 RXRA SLC6A3 SLC6A4
PR IR HP I	MOL000449	Stigmasterol	ADH1C ADRA1A ADRA1B ADRA2A ADRB1 ADRB2 AKR1B1 CHRM1 CHRM2 CHRM3 CTRB1 GABRA1 IGHG1 LTA4H MAOA MAOB NCOA1 NCOA2 NR3C2 PGR PLAU PTGS1 PTGS2 RXRA SCN5A SLC6A2 SLC6A3
RB	MOL000497	licochalcone a	ADRA1B ADRB2 AR BCL2 CA2 CALM1 CCNA2 CCND1 CDK2 CDK4 CHEK1 CHRM1 EIF6 ESR1 ESR2 FOSL2 GSK3B HSP90AA1 MAPK1 MAPK14 NCOA2 NOS2 PPARG PTGS1 PTGS2 RB1 RELA SCN5A SLC6A3 STAT3
FF	MOL000791	bicuculline	ACHE ALDH3A1 AR BMPR2 CRH FOS GABBR1 GJA1 GJB1 GNRH1 GNRHR GRIN2D GRM1 GRM5 HSP90AA1 HTR KCNH2 KDR PTGS1 PTGS2 SCN5A SLC6A2 VCP
IR	MOL000953	CLR	NCOA2 NR3C2 PGR
RB	MOL001484	Inermine	ADRA1B ADRA1D ADRB2 CALM1 CHRM1 CHRM3 HSP90AA1 HTR3A IGHG1 OPRM1 PRSS1 PTGS1 PTGS2 RXRA SCN5A
HP	MOL001645	Linoleyl acetate	NCOA2 PTGS1 PTGS2 RXRA
PR	MOL001677	asperglaucide	HTR KCNH2 PRSS1 PTGS2
PR; IR	MOL001689	acacetin	ADRB2 AR BAX BCL2 CALM1 CASP3 CASP8 CDK2 CDKN1A CHEK1 CYP19A1 DPP4 FASLG FASN HSP90AA1 NCOA1 NCOA2 NOS2 PRSS1 PTGS1 PTGS2 RELA TP63
PR	MOL001697	Sinoacutine	ACHE ADRA1A ADRA1B AR CHRM1 CHRM2 CHRM3 CHRM4 CHRM5 ESR1 ESR2 GABRA1 HTR OPRD1 OPRM1 PTGS1 PTGS2 SCN5A
IR	MOL001749	ZINC03860434	ADRB2 CHRM1 CHRM3 SCN5A
IR	MOL001755	24-Ethylcholest-4-en-3-one	NR3C2 PGR
IR	MOL001756	quindoline	MAOB NCOA2 PKIA PTGS1 PTGS2
IR	MOL001771	poriferast-5-en-3beta-ol	NCOA2 PGR
IR	MOL001774	Ineketone	NR3C2
IR	MOL001779	Sinoacutine	ACHE ADRA1B AR CALM1 CHRM1 CHRM3 CHRM5 DPP4 ESR1 ESR2 HSP90AA1 HTR NOS2 OPRD1 OPRM1 PTGS1 PTGS2 RXRA SCN5A
IR	MOL001781	Indigo	CCNA2 CDK2 PTGS1 PTGS2 RXRA
IR	MOL001782	(2Z)-2-(2-oxoindolin-3-ylidene)indolin-3-one	AR CCNA2 CDK2 CHEK1 ESR1 GABRA1 GSK3B HSP90AA1 MAPK14 NOS2 PTGS1 PTGS2 RXRA
IR	MOL001783	2-(9-((3-methyl-2-oxopent-3-en-1-yl)oxy)-2-oxo-1,2,8,9-tetrahydrofuro[2,3-h]quinolin-8-yl)propan-2-yl acetate	HSP90AA1 KCNH2 NCONA2 PRSS1 PTGS2
IR RB	MOL001792	DFV	ADRB2 ESR1 HSP90AA1 MAOB PKIA PTGS1 PTGS2 RXRA SLC6A4
IR	MOL001793	(E)-2-[(3-indole)cyanomethylene-]-3-indolinone	AR CCNA2 CDK2 CHEK1 ESR1 GSK3B HSP90AA1 MAPK14 NOS2 PTGS1 PTGS2 RXRA
IR	MOL001800	rosasterol	PGR
IR	MOL001803	Sinensetin	ACHE ADRA1B ADRB2 AR CALM1 CHEK1 DPP4 ESR2 F7 HSP90AA1 HTR KCNH2 NCOA1 NCOA2 NOS2 PRSS1 PTGS1 PTGS2 SCN5A
IR	MOL001804	Stigmasta-5,22-diene-3beta,7alpha-diol	NCOA2 PGR
IR	MOL001810	6-(3-oxoindolin-2-ylidene)indolo[2,1-b]quinazolin-12-one	ESR1 KDR PRSS1 PTGS1 PTGS2
IR	MOL001814	(E)-3-(3,5-dimethoxy-4-hydroxy-benzylidene)-2-indolinone	GABRA1 HSP90AA1 PTGS1 PTGS2 RXRA SCN5A
IR	MOL001820	(E)-3-(3,5-dimethoxy-4-hydroxyb-enzylidene)-2-indolinone	ADRB2 CHRM1 GABRA1 HSP90AA1 PTGS1 PTGS2 RXRA SCN5A
IR	MOL001828	3-[(3,5-dimethoxy-4-oxo-1-cyclohexa-2,5-dienylidene)methyl]-2,4-dihydro-1H-pyrrolo[2,1-b]quinazolin-9-one	F7 HSP90AA1 KCNH2 PRSS1 PTGS1 PTGS2 SCN5A
RB	MOL002311	Glycyrol	CCNA2 CHEK1 ESR1 GSK3B HTR KDR MAPK14 NOS2 PPARG PTGS2
RB	MOL002565	Medicarpin	ADRA1A ADRA1B ADRA1D ADRB2 CALM1 CCNA2 CDK2 CHRM1 CHRM2 CHRM3 CHRM4 CHRM5 DPP4 DRD1 ESR1 ESR2 HSP90AA1 MAPK10 NOS2 OPRD1 OPRM1 PRSS1 PTGS1 PTGS2 RXRA SCN5A SLC6A3 SLC6A4
FF	MOL003290	(3R,4R)-3,4-bis[(3,4-dimethoxyphenyl)methyl]oxolan-2-one	ADRA1B ADRA1D ADRB2 CALM1 CHRM3 ESR1 F7 HSP90AA1 KCNH2 NCOA2 PTGS2 SCN5A SLC6A3
FF	MOL003295	(+)-pinoresinol monomethyl ether	ADRA1B ADRB2 CALM1 HSP90AA1 KCNH2 NCOA1 NCOA2 PTGS1 PTGS2 RXRA RXRB SCN5A
FF	MOL003306	ACon1_001697	ADRA1B ADRB2 CALM1 HSP90AA1 KCNH2 NCOA1 NCOA2 PTGS1 PTGS2 SCN5A
FF	MOL003308	(+)-pinoresinol monomethyl ether-4-D-beta-glucoside_qt	ADRB2 CALM1 HSP90AA1 KCNH2 NCOA1 NCOA2 PTGS2 SCN5A
FF	MOL003315	3beta-Acetyl-20,25-epoxydammarane-24alpha-ol	NR3C1
FF	MOL003322	FORSYTHINOL	ADRA1B ADRB2 CALM1 HSP90AA1 KCNH2 NCOA1 NCOA2 PTGS2 SCN5A
FF	MOL003330	(-)-Phillygenin	ADRA1B ADRB2 CALM1 CHRM1 CHRM3 CHRM5 HSP90AA1 IGHG1 KCNH2 NCOA2 PTGS2 SCN5A
FF	MOL003347	hyperforin	CYP3A4 ICAM1 IL8RA NR1I2
FF	MOL003370	Onjixanthone I	CALM1 CHEK1 DPP4 ESR2 HSP90AA1 NOS2 PTGS1 PTGS2 RXRA SCN5A
RB	MOL003656	Lupiwighteone	AR CALM1 CCNA2 CDK2 CHEK1 DPP4 ESR1 ESR2 GSK3B HSP90AA1 HTR MAPK14 NCOA2 NOS2 PPARG PRSS1 PTGS2 SCN5A
RB	MOL003896	7-Methoxy-2-methyl isoflavone	ACHE ADRA1B ADRA1D ADRB1 ADRB2 AR CALM1 CCNA2 CDK2 CHEK1 CHRM1 CHRM3 CHRM5 DPP4 DRD1 ESR1 ESR2 GABRA1 GSK3B HSP90AA1 HTR IGHG1 LTA4H MAOB MAPK14 NCOA1 NCOA2 NOS2 OPRM1 PKIA PPARG PRSS1 PTGS1 PTGS2 RXRA SCN5A SLC6A3 SLC6A4
HP	MOL004598	3,5,6,7-tetramethoxy-2-(3,4,5-trimethoxyphenyl)chromone	ACHE AR CALM1 ESR1 ESR2 F7 HTR NCOA2 PRSS1 PTGS2
HP	MOL004609	Areapillin	AR CALM1 DPP4 ESR2 F7 HSP90AA1 HTR IGHG1 NCOA1 NCOA2 NOS2 PRSS1 PTGS2 SCN5A
HP	MOL004624	Longikaurin A	CHRM1 CHRM2 PRSS1
HP	MOL004653	(+)-Anomalin	DPP4 HTR KCNH2 PTGS2
HP	MOL004718	α-spinasterol	NCOA2 NR3C2 PGR
RB	MOL004805	(2S)-2-[4-hydroxy-3-(3-methylbut-2-enyl)phenyl]-8,8-dimethyl-2,3-dihydropyrano[2,3-f]chromen-4-one	AR CALM1 ESR1 ESR2 GSK3B KCNH2 MAPK14 NOS2 PPARG PTGS2
RB	MOL004806	euchrenone	BACE1 CALM1 ESR1 ESR2 KCNH2 NOS2 PTGS2 SCN5A
RB	MOL004808	glyasperin B	ACHE AR CALM1 CCNA2 CDK2 DPP4 ESR1 ESR2 F7 GSK3B HSP90AA1 HTR KDR NCOA2 NOS2 PPARG PRSS1 PTGS2
RB	MOL004810	glyasperin F	AR CALM1 CCNA2 CDK2 ESR1 ESR2 GSK3B HSP90AA1 MAPK14 NOS2 PPARG PRSS1 PTGS1 PTGS2 SCN5A
RB	MOL004811	Glyasperin C	ACHE AR CALM1 CCNA2 CDK2 CHEK1 DPP4 ESR1 ESR2 GSK3B HSP90AA1 HTR KCNH2 MAPK14 NCOA2 NOS2 PPARG PRSS1 PTGS2 RXRA SCN5A
RB	MOL004814	Isotrifoliol	AR CCNA2 CDK2 CHEK1 ESR1 ESR2 GSK3B HSP90AA1 MAPK14 NOS2 PTGS2
RB	MOL004815	(E)-1-(2,4-dihydroxyphenyl)-3-(2,2-dimethylchromen-6-yl)prop-2-en-1-one	ADRA1B AR CA2 CALM1 CCNA2 CDK2 CHEK1 ESR1 ESR2 GSK3B MAPK14 NCOA2 NOS2 PPARG PTGS1 PTGS2 RXRA SCN5A
RB	MOL004820	kanzonols W	AR CALM1 CCNA2 CDK2 CHEK1 ESR1 ESR2 GSK3B MAPK14 NCOA1 NCOA2 NOS2 PPARG PRSS1 PTGS1 PTGS2 RXRA SCN5A
RB	MOL004828	Glepidotin A	AR CALM1 CCNA2 CDK2 CHEK1 DPP4 ESR1 F7 GSK3B HSP90AA1 HTR IGHG1 KDR MAPK14 NOS2 PPARG PRSS1 PTGS1 PTGS2 RXRA SCN5A
RB	MOL004829	Glepidotin B	ADRA1B CALM1 ESR1 F7 HSP90AA1 IGHG1 NCOA1 PTGS1 PTGS2 RXRA SCN5A
RB	MOL004833	Phaseolinisoflavan	ACHE ADRA1B ADRB2 AR CALM1 CCNA2 CDK2 CHEK1 CHRM1 ESR1 ESR2 GSK3B MAPK14 NCOA1 NOS2 PPARG PRSS1 PTGS2 RXRA SCN5A
RB	MOL004835	Glypallichalcone	ADRA1B ADRB2 AR CA2 CALM1 CCNA2 CDK2 CHEK1 CHRM1 ESR1 ESR2 GSK3B HSP90AA1 LTA4H MAOB MAPK14 NCOA1 NOS2 PKIA PPARG PTGS1 PTGS2 SCN5A SLC6A3 SLC6A4
RB	MOL004838	8-(6-hydroxy-2-benzofuranyl)-2,2-dimethyl-5-chromenol	ESR1 HSP90AA1 NOS2 PTGS2 RXRA
RB	MOL004848	licochalcone G	AR CALM1 CCNA2 CDK2 ESR1 ESR2 GSK3B HSP90AA1 IGHG1 KDR MAPK14 NCOA2 NOS2 PPARG PTGS2
RB	MOL004849	3-(2,4-dihydroxyphenyl)-8-(1,1-dimethylprop-2-enyl)-7-hydroxy-5-methoxy-coumarin	AR CALM1 CDK2 CHEK1 DPP4 ESR1 ESR2 F7 GSK3B HSP90AA1 HTR KCNH2 KDR MAPK14 NCOA1 NCOA2 NOS2 PPARG PRSS1 PTGS2
RB	MOL004855	Licoricone	AR CALM1 CHEK1 ESR1 HTR KCNH2 KDR NCOA2 NOS2 PPARG PRSS1 PTGS2
RB	MOL004856	RBnin A	ACHE AR CALM1 CCNA2 CHEK1 DPP4 ESR1 ESR2 GSK3B HSP90AA1 HTR NCOA2 NOS2 PPARG PRSS1 PTGS2 SCN5A
RB	MOL004857	RBnin B	ADRA1B ADRB2 AR CALM1 CCNA2 CHEK1 DPP4 ESR1 ESR2 F7 GSK3B HSP90AA1 HTR KDR NCOA2 NOS2 PPARG PRSS1 PTGS2
RB	MOL004863	3-(3,4-dihydroxyphenyl)-5,7-dihydroxy-8-(3-methylbut-2-enyl)chromone	AR CALM1 CCNA2 CDK2 CHEK1 ESR1 GSK3B HSP90AA1 HTR MAPK14 NCOA2 NOS2 PPARG PRSS1 PTGS2
RB	MOL004866	2-(3,4-dihydroxyphenyl)-5,7-dihydroxy-6-(3-methylbut-2-enyl)chromone	ADRB2 AR CALM1 CCNA2 CDK2 CHEK1 DPP4 F7 HSP90AA1 HTR PPARG PRSS1 PTGS2 SCN5A
RB	MOL004879	Glycyrin	AR CALM1 CHEK1 DPP4 ESR1 ESR2 HTR KCNH2 KDR NCOA2 NOS2 PPARG PRSS1 PTGS2
RB	MOL004882	Licocoumarone	AR CCNA2 CDK2 ESR1 ESR2 GSK3B HSP90AA1
RB	MOL004883	Licoisoflavone	AR CALM1 CCNA2 CDK2 CHEK1 DPP4 ESR1 HSP90AA1 HTR KDR MAPK14 NCOA2 NOS2 PPARG PRSS1 PTGS2
RB	MOL004884	Licoisoflavone B	ACHE AR CALM1 CCNA2 CDK2 CHEK1 ESR1 ESR2 GSK3B HTR NOS2 PPARG PRSS1 PTGS2
RB	MOL004885	licoisoflavanone	ACHE AR CALM1 CCNA2 CDK2 ESR1 ESR2 F7 GSK3B HSP90AA1 NCOA1 NOS2 PPARG PRSS1 PTGS1 PTGS2 SCN5A
RB	MOL004891	shinpterocarpin	ADRA1B ADRA1D ADRB2 AR CALM1 CCNA2 CDK2 CHRM1 CHRM3 ESR1 ESR2 GSK3B HTR3A KCNH2 MAPK14 NCOA1 NOS2 OPRD1 OPRM1 PPARG PRSS1 PTGS1 PTGS2 RXRA RXRB SCN5A
RB	MOL004907	Glyzaglabrin	AR CCNA2 CDK2 CHEK1 DPP4 ESR1 ESR2 GSK3B HSP90AA1 MAPK14 NOS2 PPARG PRSS1 PTGS1 PTGS2
RB	MOL004908	Glabridin	ACHE ADRA1B ADRB2 AR CALM1 CCNA2 CDK2 CHEK1 CHRM1 ESR1 ESR2 GSK3B IGHG1 MAPK14 NCOA1 NCOA2 NOS2 PPARG PRSS1 PTGS2 RXRA RXRB SCN5A
RB	MOL004910	Glabranin	CALM1 ESR1 HSP90AA1 NOS2 PTGS1 PTGS2 SCN5A
RB	MOL004911	Glabrene	ADRB2 AR CALM1 CDK2 ESR1 ESR2 GSK3B HSP90AA1 MAPK14 NCOA2 NOS2 PPARG PRSS1 PTGS1 PTGS2 RXRA SCN5A
RB	MOL004912	Glabrone	ACHE AR CALM1 CCNA2 CDK2 CHEK1 DPP4 ESR1 ESR2 GSK3B HTR MAPK14 NOS2 PPARG PRSS1 PTGS1 PTGS2 RXRA SCN5A
RB	MOL004913	1,3-dihydroxy-9-methoxy-6-benzofurano[3,2-c]chromenone	CCNA2 CDK2 CHEK1 ESR1 ESR2 GSK3B HSP90AA1 MAPK14 PPARG
RB	MOL004915	Eurycarpin A	AR CALM1 CCNA2 CDK2 CHEK1 DPP4 ESR1 ESR2 GSK3B HSP90AA1 HTR MAPK14 NOS2 PPARG PRSS1 PTGS2 SCN5A
RB	MOL004941	(2R)-7-hydroxy-2-(4-hydroxyphenyl)chroman-4-one	PTGS1 ESR1 PTGS2 RXRA ADRB2 HSP90AA1 MAOB PKIA CALM1 GABRA1 SLC6A4
RB	MOL004945	(2S)-7-hydroxy-2-(4-hydroxyphenyl)-8-(3-methylbut-2-enyl)chroman-4-one	NOS2
RB	MOL004948	Isoglycyrol	AR DPP4 ESR1 GSK3B NOS2 PTGS2
RB	MOL004957	HMO	ADRB2 AR CALM1 CCNA2 CDK2 CHEK1 CHRM1 DPP4 ESR1 ESR2 GSK3B IGHG1 MAOB MAPK14 NOS2 PKIA PPARG PRSS1 PTGS1 PTGS2 RXRA SCN5A SLC6A3 SLC6A4
RB	MOL004959	1-Methoxyphaseollidin	ADRA1B ADRA1D ADRB2 AR CALM1 CCNA2 CDK2 ESR1 ESR2 GSK3B HSP90AA1 HTR KCNH2 KDR MAPK14 NCOA1 NCOA2 NOS2 PPARG PRSS1 PTGS1 PTGS2 RXRA SCN5A
RB	MOL004966	3'-Hydroxy-4'-O-Methylglabridin	ADRA1B ADRB2 AR CALM1 CCNA2 CDK2 CHEK1 ESR1 ESR2 F7 GSK3B HSP90AA1 KCNH2 KDR MAPK14 NCOA1 NCOA2 NOS2 PPARG PRSS1 PTGS1 PTGS2 SCN5A
RB	MOL004974	3'-Methoxyglabridin	ACHE ADRA1B ADRB2 AR CALM1 CCNA2 CDK2 CHEK1 ESR1 ESR2 F7 GSK3B HSP90AA1 KCNH2 MAPK14 NCOA1 NCOA2 NOS2 PPARG PRSS1 PTGS1 PTGS2 RXRA SCN5A
RB	MOL004978	2-[(3R)-8,8-dimethyl-3,4-dihydro-2H-pyrano[6,5-f]chromen-3-yl]-5-methoxyphenol	ACHE ADRA1B ADRB2 AR CALM1 CCNA2 CDK2 CHEK1 CHRM1 CHRM3 ESR1 ESR2 GSK3B KCNH2 MAPK14 NCOA1 NCOA2 NOS2 PPARG PRSS1 PTGS1 PTGS2 RXRA RXRB SCN5A SLC6A3
RB	MOL004980	Inflacoumarin A	ADRB2 AR CALM1 DPP4 ESR1 HSP90AA1 HTR NCOA2 PPARG PRSS1 PTGS1 PTGS2 SCN5A
RB	MOL004985	icos-5-enoic acid	NCOA2
RB	MOL004988	Kanzonol F	AR CALM1 ESR1 ESR2 NCOA2 PTGS2
RB	MOL004989	6-prenylated eriodictyol	CALM1 ESR1 F7 HSP90AA1 NOS2 PTGS2 SCN5A
RB	MOL004991	7-Acetoxy-2-methylisoflavone	ACHE ADRA1B ADRA1D ADRB2 AR CALM1 CDK2 CHEK1 DPP4 ESR1 GABRA1 GSK3B HSP90AA1 HTR MAPK14 NCOA2 NOS2 PPARG PRSS1 PTGS1 PTGS2 RXRA SCN5A
RB	MOL004996	gadelaidic acid	NCOA2
RB	MOL005000	RBnin G	AR CALM1 CCNA2 CHEK1 DPP4 ESR1 ESR2 GSK3B HSP90AA1 HTR MAPK14 NCOA2 NOS2 PPARG PRSS1 PTGS2
RB	MOL005001	RBnin H	AR CALM1 CCNA2 ESR1 HSP90AA1 KDR NCOA2 PRSS1 PTGS2
RB	MOL005003	Licoagrocarpin	ACHE ADRA1B ADRB2 AR CALM1 CCNA2 CDK2 CHRM1 CHRM3 CHRM5 ESR1 ESR2 GSK3B HSP90AA1 HTR KCNH2 MAPK14 NCOA2 NOS2 PPARG PRSS1 PTGS1 PTGS2 RXRA RXRB SCN5A
RB	MOL005007	Glyasperins M	ACHE AR CALM1 CCNA2 CDK2 ESR1 ESR2 F7 GSK3B HSP90AA1 KCNH2 KDR NCOA1 NCOA2 NOS2 PPARD PPARG PRSS1 PTGS1 PTGS2 SCN5A
RB	MOL005012	Licoagroisoflavone	AR CALM1 CCNA2 CDK2 CHEK1 DPP4 ESR1 ESR2 GSK3B HTR MAPK14 NOS2 PPARG PRSS1 PTGS2 SCN5A
RB	MOL005016	Odoratin	AR CALM1 CCNA2 CDK2 CHEK1 DPP4 ESR1 ESR2 GSK3B HSP90AA1 MAPK14 NCOA2 NOS2 PPARG PRSS1 PTGS1 PTGS2 RXRA SCN5A
RB	MOL005017	Phaseol	AR CCNA2 CDK2 CHEK1 ESR1 GSK3B HSP90AA1 HTR KDR MAPK14 PPARG PTGS2
RB	MOL005018	Xambioona	CALM1 ESR1 ESR2 NCOA2 NOS2 PTGS2
RB	MOL005020	dehydroglyasperins C	ADRB2 AR CALM1 CCNA2 CDK2 CHEK1 ESR1 ESR2 HSP90AA1 MAPK14 NCOA2 NOS2 PPARG PRSS1 PTGS2 SCN5A
PCRR	MOL013281	6,8-Dihydroxy-7-methoxyxanthone	ADRB2 CA2 CDK2 CHEK1 DPP4 GSK3B HSP90AA1 MAPK14 PKIA PTGS1 PTGS2
PCRR	MOL013287	Physovenine	ACHE ADRA1A ADRA1B ADRA2B ADRB2 AR CA2 CCNA2 CDK2 CHRM1 CHRM2 CHRM3 CHRNA2 DRD1 ESR1 ESR2 GABRA1 GRIA2 GSK3B HSP90AA1 HTR NOS2 OPRD1 OPRM1 PRSS1 PTGS1 PTGS2 RXRA SCN5A SLC6A2 SLC6A3 SLC6A4
PCRR	MOL013288	Picralinal	AR OPRD1 OPRM1 SCN5A

**Table 4:** Known therapeutic target genes for COVID-19

Gene	GC Id	Score	Gene	GC Id	Score
TNF	GC06P033397	33.08	ITGAL	GC16P030472	4.07
IL6	GC07P022765	31.28	STAT6	GC12M057095	4.04
CXCL8	GC04P073740	31.05	BAK1	GC06M033572	4.03
CD40LG	GC0XP136649	30.56	PIK3CG	GC07P106865	4.02
IL10	GC01M206767	30.33	FOS	GC14P075278	4.01
IFNG	GC12M068064	27.48	HELLS	GC10P094501	4
CRP	GC01M159715	25.76	CP	GC03M149162	3.96
STAT1	GC02M190964	22.73	APOA1	GC11M116835	3.95
MBL2	GC10M052760	22.1	RPS27A	GC02P055231	3.91
TP53	GC17M007661	19	CREBBP	GC16M003726	3.87
CCL2	GC17P034255	18.13	TFRC	GC03M196027	3.83
IL2	GC04M122451	17.68	LMAN1	GC18M059327	3.82
CCL5	GC17M035871	16.71	PLA2G4A	GC01P186798	3.81
IFNA1	GC09P021478	16.65	CEACAM5	GC19P041709	3.65
EGFR	GC07P055019	16.29	PRKCA	GC17P066302	3.65
CXCL10	GC04M076021	15.3	EIF2S1	GC14P067359	3.65
TGFB1	GC19M041301	14.98	CLEC12A	GC12P009951	3.61
IL1B	GC02M112829	13.78	SUMO1	GC02M202206	3.59
ACE2	GC0XM015494	12.32	CCR3	GC03P046227	3.56
CSF2	GC05P132073	11.95	UBB	GC17P016380	3.53
PPARG	GC03P012287	11.93	MAPKAPK2	GC01P206684	3.48
CCR5	GC03P046384	11.37	CD3D	GC11M118338	3.47
CXCL9	GC04M076001	11.3	CHKB	GC22M050578	3.43
GPT	GC08P144502	11.12	PPIA	GC07P044808	3.43
MAPK1	GC22M021754	11.09	RUNX1	GC21M034787	3.42
CASP3	GC04M184627	10.88	BCL2L1	GC20M031664	3.4
IFNB1	GC09M021077	10.77	GZMA	GC05P055102	3.38
ALB	GC04P073397	10.68	IRF1	GC05M132481	3.35
FGF2	GC04P122826	10.53	CD81	GC11P002377	3.35
SFTPD	GC10M079937	10.47	CST3	GC20M023608	3.29
CXCR3	GC0XM071615	10.18	PTGS1	GC09P122370	3.24
IL4	GC05P132673	10.12	F10	GC13P113122	3.22
HLA-B	GC06M031289	9.84	CBL	GC11P119206	3.18
CD79A	GC19P041877	9.73	CXCL11	GC04M076033	3.13
CXCL2	GC04M074097	9.61	MAVS	GC20P003827	3.12
ACE	GC17P063477	9.6	KPNB1	GC17P047649	3.1
TMPRSS2	GC21M041464	9.59	SLC17A5	GC06M073593	3.07
IRF3	GC19M049659	9.51	ITGA5	GC12M054396	3
MAPK3	GC16M030117	9.37	ARF1	GC01P228082	2.99
IL17A	GC06P052186	9.29	IFNL1	GC19P039296	2.97
IL5	GC05M132541	9.27	GRB2	GC17M075318	2.86
ICAM1	GC19P010270	9.22	CD3E	GC11P118304	2.84
CCL3	GC17M036088	9.2	ATF2	GC02M175072	2.78
IL13	GC05P132656	9.19	CEACAM3	GC19P041796	2.72
MAPK8	GC10P048306	9.08	HAVCR2	GC05M157063	2.7
TTR	GC18P031557	9.04	JAK1	GC01M064833	2.69
IL18	GC11M112143	8.58	NPM1	GC05P171387	2.67
ANPEP	GC15M089784	8.58	TBK1	GC12P064451	2.64
PIK3R1	GC05P068215	8.57	F11	GC04P186265	2.63
CTSL	GC09P087725	8.52	VHL	GC03P010205	2.63
CD209	GC19M007739	8.45	IL16	GC15P081159	2.59
DDX58	GC09M032455	8.2	KPNA2	GC17P068035	2.57
FURIN	GC15P090868	8.08	RELB	GC19P045002	2.57
ADA	GC20M044620	7.97	FCER2	GC19M007689	2.56
APOE	GC19P044906	7.97	PIK3CB	GC03M138652	2.55
MAPK14	GC06P046047	7.77	PRSS2	GC07P144731	2.54
DPP4	GC02M161992	7.64	RAPGEF3	GC12M047736	2.52
NFKB1	GC04P102501	7.61	BECN1	GC17M042810	2.51
HLA-A	GC06P033211	7.44	HAVCR1	GC05M157007	2.48
SERPINE1	GC07P101127	7.43	ISG15	GC01P001001	2.41
PIK3CA	GC03P179148	7.27	PML	GC15P073994	2.41
PTGS2	GC01M186640	7.24	PRKCE	GC02P045651	2.39
CD14	GC05M140631	7.16	CEACAM1	GC19M042507	2.38
MX1	GC21P041420	7.07	PIK3CD	GC01P009629	2.37
IFIH1	GC02M162267	6.99	ERN1	GC17M064039	2.37
BCL2	GC18M063123	6.96	IFITM1	GC11P000313	2.36
FCGR2A	GC01P161505	6.67	IRAK3	GC12P066188	2.35
CDK4	GC12M057743	6.64	NPTX1	GC17M080466	2.35
HSPA5	GC09M125234	6.59	HFE	GC06P026087	2.34
BAX	GC19P048954	6.53	TLR10	GC04M038773	2.33
CCL11	GC17P034285	6.47	SLC40A1	GC02M189560	2.3
CAT	GC11P034460	6.43	LCK	GC01P032251	2.29
HMOX1	GC22P035380	6.28	EIF2AK3	GC02M088637	2.27
SOD1	GC21P031659	6.25	POU5F1	GC06M031177	2.25
G6PD	GC0XM154531	6.06	VAPA	GC18P009904	2.15
CD4	GC12P006786	6.01	CARD9	GC09M136361	2.15
TF	GC03P133666	5.96	TRIM25	GC17M056836	2.13
CTRL	GC16M067927	5.95	HNRNPA1	GC12P054280	2.05
IL1A	GC02M112773	5.93	CCND3	GC06M041934	1.99
PIK3C2A	GC11M017165	5.92	MYOM2	GC08P002045	1.97
PARP1	GC01M226360	5.91	PRKRA	GC02M178431	1.96
RELA	GC11M065653	5.89	SOCS3	GC17M078356	1.95
NOS2	GC17M027756	5.85	LCN1	GC09P135521	1.91
EIF2AK2	GC02M037099	5.83	EIF4E	GC04M098871	1.91
GAPDH	GC12P006630	5.81	ICAM2	GC17M064002	1.89
NOS3	GC07P150990	5.77	BST2	GC19M017403	1.88
CTSB	GC08M011842	5.72	IFITM2	GC11P000300	1.87
CCL4	GC17P036103	5.69	KPNA4	GC03M160494	1.83
CASP8	GC02P201233	5.65	DROSHA	GC05M031401	1.78
ANXA5	GC04M121667	5.59	USP7	GC16M008892	1.78
F8	GC0XM154835	5.58	CD46	GC01P207752	1.74
CREB1	GC02P207529	5.55	AHSG	GC03P186612	1.73
SH2D3A	GC19M006752	5.54	BAG3	GC10P119651	1.72
HLA-DRB1	GC06M032578	5.48	TMPRSS11A	GC04M067909	1.69
TMPRSS11D	GC04M067820	5.38	APOD	GC03M195568	1.66
BMP6	GC06P007726	5.32	PRKCB	GC16P023872	1.64
SMAD3	GC15P067063	5.2	RHOB	GC02P020447	1.64
MASP2	GC01M011026	5.13	ITGA6	GC02P172427	1.63
IFITM3	GC11M000319	5.11	STAT2	GC12M056341	1.62
HLA-C	GC06M031272	5.11	CALM1	GC14P090396	1.61
BAD	GC11M064273	5.04	OAS1	GC12P112906	1.6
CANX	GC05P179678	4.97	BCL2L2	GC14P025033	1.6
MCL1	GC01M150673	4.77	IFI27	GC14P094104	1.6
CCL7	GC17P034270	4.71	PSMC6	GC14P052707	1.55
CASP6	GC04M109688	4.7	TFR2	GC07M100620	1.5
EGR1	GC05P138465	4.66	SPI1	GC11M059694	1.45
ITGB1	GC10M032900	4.64	IGKC	GC02M089081	1.44
RNASE3	GC14P020891	4.63	PHB2	GC12M006965	1.44
STING1	GC05M139476	4.5	CD151	GC11P000883	1.42
CD34	GC01M207880	4.48	ITGA1	GC05P052788	1.42
DUSP1	GC05M172768	4.42	FAH	GC15P080152	1.4
RB1	GC13P048303	4.41	NUDT2	GC09P034329	1.36
ADAM17	GC02M009488	4.4	AQP1	GC07P030911	1.35
HSPB1	GC07P076302	4.33	TMPRSS13	GC11M117900	1.32
EEF1A1	GC06M073515	4.33	CD3G	GC11P118344	1.28
TOLLIP	GC11M001274	4.31	PCSK5	GC09P075890	1.23
CCR1	GC03M046218	4.3	CBLB	GC03M105655	1.21
EZR	GC06M158765	4.27	TMEM233	GC12P119594	1.18
LCN2	GC09P128149	4.26	ANXA11	GC10M080150	1.13
TRAF3	GC14P104312	4.23	CLEC4D	GC12P008509	1.1
SMAD7	GC18M048919	4.18	NMRAL1	GC16M004461	1.07
TXN	GC09M110243	4.17	HPGDS	GC04M094298	0.84
ICAM3	GC19M010335	4.15	SLC39A14	GC08P022367	0.83
VCP	GC09M035056	4.15	OR8U9	GC11Pi00193	0.35
NLRP12	GC19M053793	4.14	C8G	GC09P136944	0.31
ANXA2	GC15M060347	4.12			

**Table 5:** The data of top twenty GO terms including BP, CC, MF

GO category	ID	Description	*P*-value	*P*.adjust	Count
BP	GO:0032496	response to lipopolysaccharide	1.27E-17	2.31E-14	13
BP	GO:0002237	response to molecule of bacterial origin	2.10E-17	2.31E-14	13
BP	GO:0071222	cellular response to lipopolysaccharide	1.39E-12	1.02E-09	9
BP	GO:0071219	cellular response to molecule of bacterial origin	1.89E-12	1.04E-09	9
BP	GO:0071216	cellular response to biotic stimulus	4.96E-12	2.18E-09	9
BP	GO:2001234	negative regulation of apoptotic signaling pathway	1.96E-10	7.20E-08	8
BP	GO:0010038	response to metal ion	2.37E-10	7.45E-08	9
BP	GO:0048545	response to steroid hormone	3.89E-10	1.07E-07	9
BP	GO:0022407	regulation of cell-cell adhesion	5.82E-10	1.42E-07	9
BP	GO:0048608	reproductive structure development	1.05E-09	2.23E-07	9
BP	GO:0061458	reproductive system development	1.12E-09	2.23E-07	9
BP	GO:0009314	response to radiation	1.48E-09	2.65E-07	9
BP	GO:0006979	response to oxidative stress	1.57E-09	2.65E-07	9
BP	GO:0042110	T cell activation	2.01E-09	3.16E-07	9
BP	GO:0034612	response to tumor necrosis factor	2.19E-09	3.22E-07	8
BP	GO:0034349	glial cell apoptotic process	2.37E-09	3.25E-07	4
BP	GO:0002573	myeloid leukocyte differentiation	3.55E-09	4.59E-07	7
BP	GO:0070997	neuron death	5.17E-09	6.31E-07	8
BP	GO:0097191	extrinsic apoptotic signaling pathway	6.78E-09	7.86E-07	7
BP	GO:0070482	response to oxygen levels	1.36E-08	1.50E-06	8
CC	GO:0045121	membrane raft	1.27E-06	6.17E-05	6
CC	GO:0098857	membrane microdomain	1.30E-06	6.17E-05	6
CC	GO:0098589	membrane region	1.61E-06	6.17E-05	6
CC	GO:0005741	mitochondrial outer membrane	4.97E-05	0.001243834	4
CC	GO:0005667	transcription factor complex	5.41E-05	0.001243834	5
CC	GO:0031968	organelle outer membrane	7.97E-05	0.001361141	4
CC	GO:0019867	outer membrane	8.29E-05	0.001361141	4
CC	GO:0046930	pore complex	0.000324441	0.004663842	2
CC	GO:1904813	ficolin-1-rich granule lumen	0.000392171	0.005011074	3
CC	GO:0005819	spindle	0.000640704	0.007368092	4
CC	GO:0090575	RNA polymerase II transcription factor complex	0.000869852	0.009093909	3
CC	GO:0000307	cyclin-dependent protein kinase holoenzyme complex	0.001089346	0.010439568	2
CC	GO:0101002	ficolin-1-rich granule	0.001253428	0.011088015	3
CC	GO:0044798	nuclear transcription factor complex	0.001590231	0.013062616	3
CC	GO:0005901	caveola	0.003891901	0.029837911	2
CC	GO:1902554	serine/threonine protein kinase complex	0.004688015	0.03369511	2
CC	GO:0035578	azurophil granule lumen	0.005004373	0.03385311	2
CC	GO:0034774	secretory granule lumen	0.005946804	0.035510845	3
CC	GO:1904949	ATPase complex	0.006127975	0.035510845	2
CC	GO:0060205	cytoplasmic vesicle lumen	0.006856895	0.035510845	3
MF	GO:0051400	BH domain binding	2.29E-07	1.91E-05	3
MF	GO:0070513	death domain binding	2.29E-07	1.91E-05	3
MF	GO:0019902	phosphatase binding	3.42E-06	0.000170898	5
MF	GO:0033613	activating transcription factor binding	4.09E-06	0.000170898	4
MF	GO:0002020	protease binding	2.08E-05	0.000629425	4
MF	GO:0005126	cytokine receptor binding	2.82E-05	0.000629425	5
MF	GO:0019903	protein phosphatase binding	2.96E-05	0.000629425	4
MF	GO:0031625	ubiquitin protein ligase binding	3.02E-05	0.000629425	5
MF	GO:0044389	ubiquitin-like protein ligase binding	4.02E-05	0.000746042	5
MF	GO:0004707	MAP kinase activity	0.000145644	0.00243226	2
MF	GO:0097153	cysteine-type endopeptidase activity involved in apoptotic process	0.000167918	0.002549304	2
MF	GO:0004708	MAP kinase kinase activity	0.000191755	0.002668588	2
MF	GO:0005123	death receptor binding	0.00021715	0.002789547	2
MF	GO:0020037	heme binding	0.000687548	0.008201468	3
MF	GO:0097718	disordered domain specific binding	0.000832459	0.00883078	2
MF	GO:0046906	tetrapyrrole binding	0.000846063	0.00883078	3
MF	GO:0001085	RNA polymerase II transcription factor binding	0.001026133	0.009707469	3
MF	GO:0016248	channel inhibitor activity	0.001103999	0.009707469	2
MF	GO:0016705	oxidoreductase activity, acting on paired donors, with incorporation or reduction of molecular oxygen	0.001104443	0.009707469	3
MF	GO:0004712	protein serine/threonine/tyrosine kinase activity	0.001412492	0.011727316	2

**Table 6:** The data of top twenty KEGG pathway

ID	Description	*P*-value	*P*.adjust	Count
hsa05167	Kaposi sarcoma-associated herpesvirus infection	5.39E-16	8.46E-14	13
hsa04933	AGE-RAGE signaling pathway in diabetic complications	1.13E-15	8.85E-14	11
hsa05163	Human cytomegalovirus infection	6.58E-15	3.45E-13	13
hsa04657	IL-17 signaling pathway	4.27E-14	1.68E-12	10
hsa05161	Hepatitis B	2.60E-13	6.81E-12	11
hsa04668	TNF signaling pathway	2.60E-13	6.81E-12	10
hsa05164	Influenza A	1.78E-11	4.00E-10	10
hsa05133	Pertussis	2.57E-11	5.04E-10	8
hsa05152	Tuberculosis	3.16E-11	5.50E-10	10
hsa05169	Epstein-Barr virus infection	9.46E-11	1.48E-09	10
hsa05170	Human immunodeficiency virus 1 infection	1.60E-10	2.29E-09	10
hsa05142	Chagas disease (American trypanosomiasis)	2.86E-10	3.74E-09	8
hsa05140	Leishmaniasis	1.57E-09	1.90E-08	7
hsa04210	Apoptosis	2.89E-09	3.24E-08	8
hsa05162	Measles	3.24E-09	3.40E-08	8
hsa05132	Salmonella infection	4.64E-09	4.55E-08	9
hsa01522	Endocrine resistance	8.69E-09	8.03E-08	7
hsa04625	C-type lectin receptor signaling pathway	1.32E-08	1.15E-07	7
hsa05145	Toxoplasmosis	2.22E-08	1.83E-07	7
hsa05130	Pathogenic Escherichia coli infection	6.53E-08	5.13E-07	8

**Table 7:** The functional annotation clustering of CGs

Annotation Cluster	Term	Count	*P*-value
Annotation Cluster 1 (Score:6.04)	Asthma|Bronchiolitis, Viral|Respiratory Syncytial Virus Infections	7	8.50E-07
	respiratory syncytial virus bronchiolitis	7	8.50E-07
	Bronchiolitis, Viral|Respiratory Syncytial Virus Infections	7	1.04E-06
Annotation Cluster 2 (Score:4.91)	Coronary Artery Disease|Inflammation	5	5.45E-07
	non-Hodgkin lymphoma	4	1.90E-06
	Recurrence|Venous Thromboembolism	5	2.48E-06
	Arthritis	5	2.66E-06
	Brain Ischemia|Hypertension|Osteoporosis|Stroke	5	3.69E-06
	diabetes, type 1	6	1.38E-05
	melanoma	5	2.10E-05
	Inflammation|Venous Thromboembolism	4	2.24E-05
	Chlamydia Infections|Inflammation|Trachoma	4	2.24E-05
	Brain Ischemia|Inflammation|Stroke	4	2.24E-05
	Pre-Eclampsia	4	3.32E-04
	Migraine Disorders	4	4.52E-04
Annotation Cluster 3 (Score:4.89)	Chorioamnionitis|Fetal Membranes, Premature Rupture|Infection of amniotic sac and membranes	7	4.94E-07
	Chorioamnionitis|Fetal Membranes, Premature Rupture|Infection of amniotic sac and membranes|Obstetric Labor, Premature|Pre-Eclampsia|Premature Birth	7	5.10E-07
	Coronary Artery Disease	7	1.56E-04
	Alzheimer's disease	8	7.10E-04
Annotation Cluster 4 (Score:4.44)	Hodgkin Disease|Inflammation	4	3.40E-06
	Sarcoidosis	5	4.99E-06
	Adenocarcinoma|Stomach Neoplasms	4	5.74E-05
	kidney failure, chronic	5	2.02E-04
	esophageal cancer	4	3.20E-04
Annotation Cluster 5 (Score:4.26)	Lymphoma, Non-Hodgkin|Lymphoma, Non-Hodgkin's	5	3.30E-05
	Leukemia, Myelogenous, Chronic, BCR-ABL Positive|Neovascularization, Pathologic	4	4.02E-05
	Leukemia, Myelogenous, Chronic, BCR-ABL Positive	4	1.29E-04
Annotation Cluster 6 (Score:4.09)	Tuberculosis, Pulmonary	5	2.31E-06
	systemic lupus erythematosus	5	8.88E-05
	hepatitis C, chronic	4	3.56E-04
	Tuberculosis	4	6.16E-04
Annotation Cluster 7 (Score:4.04)	Helicobacter Infections|Inflammation|Precancerous Conditions|Stomach Neoplasms	4	9.23E-07
	Stomach Neoplasms	5	3.54E-05
	patent ductus arteriosus	5	6.13E-05
	Cystic Fibrosis	4	1.29E-04
	stomach cancer	4	5.46E-04
	rheumatoid arthritis	4	0.003880586
Annotation Cluster 8 (Score:3.94)	Infection|Inflammation|Premature Birth	5	5.26E-05
	Inflammation|Premature Birth	5	5.77E-05
	Connective Tissue Diseases|Fetal Diseases|Inflammation|Musculoskeletal Diseases|Pregnancy Complications, Hematologic|Premature Birth|Skin Diseases	5	5.77E-05
	Asthma	4	9.96E-04
Annotation Cluster 9 (Score:3.84)	Atherosclerosis	7	2.07E-05
	Myocardial Infarction	7	2.05E-04
	Alzheimer's disease	8	7.10E-04
Annotation Cluster 10 (Score:3.78)	Brain Ischemia|Stroke	5	1.86E-05
	Peripheral Vascular Diseases	4	4.02E-05
	Cardiovascular Diseases	5	2.60E-04
	Hypercholesterolemia|LDLC levels	4	0.004054968
Annotation Cluster 11 (Score:3.55)	Restenosis	4	1.81E-04
	Arthritis, Rheumatoid|Rheumatoid Arthritis	5	1.98E-04
	Endometriosis	4	6.16E-04
Annotation Cluster 12 (Score:3.28)	Alcoholism|Liver Cirrhosis, Alcoholic	3	2.12E-04
	Esophageal Neoplasms|Hyperglycemia|Oesophageal neoplasm	3	2.12E-04
	Biliary Tract Neoplasms|Inflammation	3	5.66E-04
	Arthritis, Psoriatic|Psoriatic arthropathy	3	6.21E-04
	cardiovascular	3	0.002488197
Annotation Cluster 13 (Score:3.18)	Otitis Media|Recurrence	3	2.47E-04
	Brucellosis	3	5.12E-04
	Graft vs Host Disease|Hematologic Neoplasms|Neoplasm Recurrence, Local	3	5.12E-04
	Kawasaki disease	3	6.21E-04
	Atopy	3	0.003077333
Annotation Cluster 14 (Score:2.82)	Atherosclerosis|Inflammation|Retinal Vein Occlusion	3	1.23E-04
	Dermatitis, Atopic|Eczema allergic	3	7.41E-04
	juvenile arthritis	3	0.001084437
	graft-versus-host disease	3	0.002834545
	Graft vs Host Disease	3	0.005354403
	hepatitis C	3	0.008600238
Annotation Cluster 15 (Score:2.80)	Uveitis, Anterior	3	1.23E-04
	Pancreatitis, Chronic	3	8.05E-04
	stroke, ischemic	3	0.004142278
	Glomerulonephritis, IGA	3	0.016033469
Annotation Cluster 16 (Score:2.76)	giant cell arteritis	3	5.12E-04
	Malaria, Falciparum	3	0.002163435
	Malaria	3	0.004882851
Annotation Cluster 17 (Score:2.72)	Cardiovascular Diseases|Inflammation	3	1.50E-04
	skin cancer, non-melanoma	3	0.001084437
	Adenoma|Colorectal Neoplasms	3	0.002954755
	Depression	3	0.028362664
Annotation Cluster 18 (Score:2.68)	Endometriosis|Uterine Diseases	3	1.50E-04
	Hepatitis B, Chronic	3	0.006357795
	Pulmonary Disease, Chronic Obstructive	3	0.009415863
Annotation Cluster 19 (Score:2.63)	respiratory syncytial virus	3	3.68E-04
	Q fever	3	4.61E-04
	Graves' disease|Graves' disease	3	0.001490775
	Graves' disease	3	0.001579503
	Diabetes Mellitus, Insulin-Dependent|Diabetes Mellitus, Type 1	3	0.006532757
	Premature Birth	3	0.01182929
	Kidney Diseases	3	0.012294446
Annotation Cluster 20 (Score:2.47)	Carcinoma, Squamous Cell|Mouth Neoplasms	3	0.001084437
	Helicobacter Infections|Stomach Neoplasms	3	0.002377534
	Precursor Cell Lymphoblastic Leukemia-Lymphoma	3	0.015509841

## Abbreviations

ACE2: Angiotensin converting enzyme 2
ARDS: acute respiratory distress syndrome
BBB: blood-brain barrier
BC: Betweenness Centrality
BP: biological processes
Caco-2: Caco-2 permeability
CC: cellular components
CC: Colseness Centrality
CG: candidate genes
DC: Degree Centrality
DL: drug-likeness (DL)
EC: Eigenvector Centrality
FF: Forsythiae Fructus
GO: Gene Ontology
HP: Herba Patriniae
I: licorice
IR: Isatidis Radix
KEGG: Kyoto Encyclopedia of Genes and Genomes
LAC: Local average connectivity-based method
LHQWG: LianHua QingWen granules
MF: molecular functions
NC: Network Centrality
NCP: Novel Coronavirus Pneumonia
OB: oral bioavailability
PCRR: Polygoni Cuspidati Rhizoma Et Radix
PPI: protein-protein interaction
PR: Phragmitis Rhizoma
RB: Radix Bupleuri
SFJDC: ShuFeng JieDu capsule
SARS-COV-2: Severe Acute Respiratory Syndrome-Coronavirus-2
TCM: Traditional Chinese Medicine
TCMSP: Traditional Chinese Medicine Systems Pharmacology Database and Analysis Platform
VH: Verbenae Herb
